# Search for new phenomena in final states with an energetic jet and large missing transverse momentum in *pp* collisions at $\sqrt{s}=8~$TeV with the ATLAS detector

**DOI:** 10.1140/epjc/s10052-015-3517-3

**Published:** 2015-07-01

**Authors:** G. Aad, B. Abbott, J. Abdallah, S. Abdel Khalek, O. Abdinov, R. Aben, B. Abi, M. Abolins, O. S. AbouZeid, H. Abramowicz, H. Abreu, R. Abreu, Y. Abulaiti, B. S. Acharya, L. Adamczyk, D. L. Adams, J. Adelman, S. Adomeit, T. Adye, T. Agatonovic-Jovin, J. A. Aguilar-Saavedra, M. Agustoni, S. P. Ahlen, F. Ahmadov, G. Aielli, H. Akerstedt, T. P. A. Åkesson, G. Akimoto, A. V. Akimov, G. L. Alberghi, J. Albert, S. Albrand, M. J. Alconada Verzini, M. Aleksa, I. N. Aleksandrov, C. Alexa, G. Alexander, G. Alexandre, T. Alexopoulos, M. Alhroob, G. Alimonti, L. Alio, J. Alison, B. M. M. Allbrooke, L. J. Allison, P. P. Allport, A. Aloisio, A. Alonso, F. Alonso, C. Alpigiani, A. Altheimer, B. Alvarez Gonzalez, M. G. Alviggi, K. Amako, Y. Amaral Coutinho, C. Amelung, D. Amidei, S. P. Amor Dos Santos, A. Amorim, S. Amoroso, N. Amram, G. Amundsen, C. Anastopoulos, L. S. Ancu, N. Andari, T. Andeen, C. F. Anders, G. Anders, K. J. Anderson, A. Andreazza, V. Andrei, X. S. Anduaga, S. Angelidakis, I. Angelozzi, P. Anger, A. Angerami, F. Anghinolfi, A. V. Anisenkov, N. Anjos, A. Annovi, M. Antonelli, A. Antonov, J. Antos, F. Anulli, M. Aoki, L. Aperio Bella, G. Arabidze, Y. Arai, J. P. Araque, A. T. H. Arce, F. A. Arduh, J-F. Arguin, S. Argyropoulos, M. Arik, A. J. Armbruster, O. Arnaez, V. Arnal, H. Arnold, M. Arratia, O. Arslan, A. Artamonov, G. Artoni, S. Asai, N. Asbah, A. Ashkenazi, B. Åsman, L. Asquith, K. Assamagan, R. Astalos, M. Atkinson, N. B. Atlay, B. Auerbach, K. Augsten, M. Aurousseau, G. Avolio, B. Axen, M. K. Ayoub, G. Azuelos, M. A. Baak, A. E. Baas, C. Bacci, H. Bachacou, K. Bachas, M. Backes, M. Backhaus, P. Bagiacchi, P. Bagnaia, Y. Bai, T. Bain, J. T. Baines, O. K. Baker, P. Balek, T. Balestri, F. Balli, E. Banas, Sw. Banerjee, A. A. E. Bannoura, H. S. Bansil, L. Barak, S. P. Baranov, E. L. Barberio, D. Barberis, M. Barbero, T. Barillari, M. Barisonzi, T. Barklow, N. Barlow, S. L. Barnes, B. M. Barnett, R. M. Barnett, Z. Barnovska, A. Baroncelli, G. Barone, A. J. Barr, F. Barreiro, J. Barreiro Guimarães da Costa, R. Bartoldus, A. E. Barton, P. Bartos, A. Bassalat, A. Basye, R. L. Bates, S. J. Batista, J. R. Batley, M. Battaglia, M. Bauce, F. Bauer, H. S. Bawa, J. B. Beacham, M. D. Beattie, T. Beau, P. H. Beauchemin, R. Beccherle, P. Bechtle, H. P. Beck, K. Becker, S. Becker, M. Beckingham, C. Becot, A. J. Beddall, A. Beddall, V. A. Bednyakov, C. P. Bee, L. J. Beemster, T. A. Beermann, M. Begel, K. Behr, C. Belanger-Champagne, P. J. Bell, W. H. Bell, G. Bella, L. Bellagamba, A. Bellerive, M. Bellomo, K. Belotskiy, O. Beltramello, O. Benary, D. Benchekroun, M. Bender, K. Bendtz, N. Benekos, Y. Benhammou, E. Benhar Noccioli, J. A. Benitez Garcia, D. P. Benjamin, J. R. Bensinger, S. Bentvelsen, L. Beresford, M. Beretta, D. Berge, E. Bergeaas Kuutmann, N. Berger, F. Berghaus, J. Beringer, C. Bernard, N. R. Bernard, C. Bernius, F. U. Bernlochner, T. Berry, P. Berta, C. Bertella, G. Bertoli, F. Bertolucci, C. Bertsche, D. Bertsche, M. I. Besana, G. J. Besjes, O. Bessidskaia Bylund, M. Bessner, N. Besson, C. Betancourt, S. Bethke, A. J. Bevan, W. Bhimji, R. M. Bianchi, L. Bianchini, M. Bianco, O. Biebel, S. P. Bieniek, M. Biglietti, J. Bilbao De Mendizabal, H. Bilokon, M. Bindi, S. Binet, A. Bingul, C. Bini, C. W. Black, J. E. Black, K. M. Black, D. Blackburn, R. E. Blair, J.-B. Blanchard, J. E. Blanco, T. Blazek, I. Bloch, C. Blocker, W. Blum, U. Blumenschein, G. J. Bobbink, V. S. Bobrovnikov, S. S. Bocchetta, A. Bocci, C. Bock, C. R. Boddy, M. Boehler, J. A. Bogaerts, A. G. Bogdanchikov, C. Bohm, V. Boisvert, T. Bold, V. Boldea, A. S. Boldyrev, M. Bomben, M. Bona, M. Boonekamp, A. Borisov, G. Borissov, S. Borroni, J. Bortfeldt, V. Bortolotto, K. Bos, D. Boscherini, M. Bosman, H. Boterenbrood, J. Boudreau, J. Bouffard, E. V. Bouhova-Thacker, D. Boumediene, C. Bourdarios, N. Bousson, S. Boutouil, A. Boveia, J. Boyd, I. R. Boyko, I. Bozic, J. Bracinik, A. Brandt, G. Brandt, O. Brandt, U. Bratzler, B. Brau, J. E. Brau, H. M. Braun, S. F. Brazzale, K. Brendlinger, A. J. Brennan, L. Brenner, R. Brenner, S. Bressler, K. Bristow, T. M. Bristow, D. Britton, F. M. Brochu, I. Brock, R. Brock, J. Bronner, G. Brooijmans, T. Brooks, W. K. Brooks, J. Brosamer, E. Brost, J. Brown, P. A. Bruckman de Renstrom, D. Bruncko, R. Bruneliere, A. Bruni, G. Bruni, M. Bruschi, L. Bryngemark, T. Buanes, Q. Buat, F. Bucci, P. Buchholz, A. G. Buckley, S. I. Buda, I. A. Budagov, F. Buehrer, L. Bugge, M. K. Bugge, O. Bulekov, H. Burckhart, S. Burdin, B. Burghgrave, S. Burke, I. Burmeister, E. Busato, D. Büscher, V. Büscher, P. Bussey, C. P. Buszello, J. M. Butler, A. I. Butt, C. M. Buttar, J. M. Butterworth, P. Butti, W. Buttinger, A. Buzatu, S. Cabrera Urbán, D. Caforio, O. Cakir, P. Calafiura, A. Calandri, G. Calderini, P. Calfayan, L. P. Caloba, D. Calvet, S. Calvet, R. Camacho Toro, S. Camarda, D. Cameron, L. M. Caminada, R. Caminal Armadans, S. Campana, M. Campanelli, A. Campoverde, V. Canale, A. Canepa, M. Cano Bret, J. Cantero, R. Cantrill, T. Cao, M. D. M. Capeans Garrido, I. Caprini, M. Caprini, M. Capua, R. Caputo, R. Cardarelli, T. Carli, G. Carlino, L. Carminati, S. Caron, E. Carquin, G. D. Carrillo-Montoya, J. R. Carter, J. Carvalho, D. Casadei, M. P. Casado, M. Casolino, E. Castaneda-Miranda, A. Castelli, V. Castillo Gimenez, N. F. Castro, P. Catastini, A. Catinaccio, J. R. Catmore, A. Cattai, G. Cattani, J. Caudron, V. Cavaliere, D. Cavalli, M. Cavalli-Sforza, V. Cavasinni, F. Ceradini, B. Cerio, K. Cerny, A. S. Cerqueira, A. Cerri, L. Cerrito, F. Cerutti, M. Cerv, A. Cervelli, S. A. Cetin, A. Chafaq, D. Chakraborty, I. Chalupkova, P. Chang, B. Chapleau, J. D. Chapman, D. Charfeddine, D. G. Charlton, C. C. Chau, C. A. Chavez Barajas, S. Cheatham, A. Chegwidden, S. Chekanov, S. V. Chekulaev, G. A. Chelkov, M. A. Chelstowska, C. Chen, H. Chen, K. Chen, L. Chen, S. Chen, X. Chen, Y. Chen, H. C. Cheng, Y. Cheng, A. Cheplakov, E. Cheremushkina, R. Cherkaoui El Moursli, V. Chernyatin, E. Cheu, L. Chevalier, V. Chiarella, J. T. Childers, A. Chilingarov, G. Chiodini, A. S. Chisholm, R. T. Chislett, A. Chitan, M. V. Chizhov, S. Chouridou, B. K. B. Chow, D. Chromek-Burckhart, M. L. Chu, J. Chudoba, J. J. Chwastowski, L. Chytka, G. Ciapetti, A. K. Ciftci, D. Cinca, V. Cindro, A. Ciocio, Z. H. Citron, M. Ciubancan, A. Clark, P. J. Clark, R. N. Clarke, W. Cleland, C. Clement, Y. Coadou, M. Cobal, A. Coccaro, J. Cochran, L. Coffey, J. G. Cogan, B. Cole, S. Cole, A. P. Colijn, J. Collot, T. Colombo, G. Compostella, P. Conde Muiño, E. Coniavitis, S. H. Connell, I. A. Connelly, S. M. Consonni, V. Consorti, S. Constantinescu, C. Conta, G. Conti, F. Conventi, M. Cooke, B. D. Cooper, A. M. Cooper-Sarkar, K. Copic, T. Cornelissen, M. Corradi, F. Corriveau, A. Corso-Radu, A. Cortes-Gonzalez, G. Cortiana, G. Costa, M. J. Costa, D. Costanzo, D. Côté, G. Cottin, G. Cowan, B. E. Cox, K. Cranmer, G. Cree, S. Crépé-Renaudin, F. Crescioli, W. A. Cribbs, M. Crispin Ortuzar, M. Cristinziani, V. Croft, G. Crosetti, T. Cuhadar Donszelmann, J. Cummings, M. Curatolo, C. Cuthbert, H. Czirr, P. Czodrowski, S. D’Auria, M. D’Onofrio, M. J. Da Cunha Sargedas De Sousa, C. Da Via, W. Dabrowski, A. Dafinca, T. Dai, O. Dale, F. Dallaire, C. Dallapiccola, M. Dam, J. R. Dandoy, A. C. Daniells, M. Danninger, M. Dano Hoffmann, V. Dao, G. Darbo, S. Darmora, J. Dassoulas, A. Dattagupta, W. Davey, C. David, T. Davidek, E. Davies, M. Davies, O. Davignon, P. Davison, Y. Davygora, E. Dawe, I. Dawson, R. K. Daya-Ishmukhametova, K. De, R. de Asmundis, S. De Castro, S. De Cecco, N. De Groot, P. de Jong, H. De la Torre, F. De Lorenzi, L. De Nooij, D. De Pedis, A. De Salvo, U. De Sanctis, A. De Santo, A. De Simone, J. B. De Vivie De Regie, W. J. Dearnaley, R. Debbe, C. Debenedetti, D. V. Dedovich, I. Deigaard, J. Del Peso, T. Del Prete, D. Delgove, F. Deliot, C. M. Delitzsch, M. Deliyergiyev, A. Dell’Acqua, L. Dell’Asta, M. Dell’Orso, M. Della Pietra, D. della Volpe, M. Delmastro, P. A. Delsart, C. Deluca, D. A. DeMarco, S. Demers, M. Demichev, A. Demilly, S. P. Denisov, D. Derendarz, J. E. Derkaoui, F. Derue, P. Dervan, K. Desch, C. Deterre, P. O. Deviveiros, A. Dewhurst, S. Dhaliwal, A. Di Ciaccio, L. Di Ciaccio, A. Di Domenico, C. Di Donato, A. Di Girolamo, B. Di Girolamo, A. Di Mattia, B. Di Micco, R. Di Nardo, A. Di Simone, R. Di Sipio, D. Di Valentino, C. Diaconu, M. Diamond, F. A. Dias, M. A. Diaz, E. B. Diehl, J. Dietrich, T. A. Dietzsch, S. Diglio, A. Dimitrievska, J. Dingfelder, F. Dittus, F. Djama, T. Djobava, J. I. Djuvsland, M. A. B. do Vale, D. Dobos, M. Dobre, C. Doglioni, T. Doherty, T. Dohmae, J. Dolejsi, Z. Dolezal, B. A. Dolgoshein, M. Donadelli, S. Donati, P. Dondero, J. Donini, J. Dopke, A. Doria, M. T. Dova, A. T. Doyle, M. Dris, E. Dubreuil, E. Duchovni, G. Duckeck, O. A. Ducu, D. Duda, A. Dudarev, L. Duflot, L. Duguid, M. Dührssen, M. Dunford, H. Duran Yildiz, M. Düren, A. Durglishvili, D. Duschinger, M. Dwuznik, M. Dyndal, K. M. Ecker, W. Edson, N. C. Edwards, W. Ehrenfeld, T. Eifert, G. Eigen, K. Einsweiler, T. Ekelof, M. El Kacimi, M. Ellert, S. Elles, F. Ellinghaus, A. A. Elliot, N. Ellis, J. Elmsheuser, M. Elsing, D. Emeliyanov, Y. Enari, O. C. Endner, M. Endo, R. Engelmann, J. Erdmann, A. Ereditato, D. Eriksson, G. Ernis, J. Ernst, M. Ernst, S. Errede, E. Ertel, M. Escalier, H. Esch, C. Escobar, B. Esposito, A. I. Etienvre, E. Etzion, H. Evans, A. Ezhilov, L. Fabbri, G. Facini, R. M. Fakhrutdinov, S. Falciano, R. J. Falla, J. Faltova, Y. Fang, M. Fanti, A. Farbin, A. Farilla, T. Farooque, S. Farrell, S. M. Farrington, P. Farthouat, F. Fassi, P. Fassnacht, D. Fassouliotis, A. Favareto, L. Fayard, P. Federic, O. L. Fedin, W. Fedorko, S. Feigl, L. Feligioni, C. Feng, E. J. Feng, H. Feng, A. B. Fenyuk, P. Fernandez Martinez, S. Fernandez Perez, S. Ferrag, J. Ferrando, A. Ferrari, P. Ferrari, R. Ferrari, D. E. Ferreira de Lima, A. Ferrer, D. Ferrere, C. Ferretti, A. Ferretto Parodi, M. Fiascaris, F. Fiedler, A. Filipčič, M. Filipuzzi, F. Filthaut, M. Fincke-Keeler, K. D. Finelli, M. C. N. Fiolhais, L. Fiorini, A. Firan, A. Fischer, C. Fischer, J. Fischer, W. C. Fisher, E. A. Fitzgerald, M. Flechl, I. Fleck, P. Fleischmann, S. Fleischmann, G. T. Fletcher, G. Fletcher, T. Flick, A. Floderus, L. R. Flores Castillo, M. J. Flowerdew, A. Formica, A. Forti, D. Fournier, H. Fox, S. Fracchia, P. Francavilla, M. Franchini, D. Francis, L. Franconi, M. Franklin, M. Fraternali, D. Freeborn, S. T. French, F. Friedrich, D. Froidevaux, J. A. Frost, C. Fukunaga, E. Fullana Torregrosa, B. G. Fulsom, J. Fuster, C. Gabaldon, O. Gabizon, A. Gabrielli, A. Gabrielli, S. Gadatsch, S. Gadomski, G. Gagliardi, P. Gagnon, C. Galea, B. Galhardo, E. J. Gallas, B. J. Gallop, P. Gallus, G. Galster, K. K. Gan, J. Gao, Y. S. Gao, F. M. Garay Walls, F. Garberson, C. García, J. E. García Navarro, M. Garcia-Sciveres, R. W. Gardner, N. Garelli, V. Garonne, C. Gatti, G. Gaudio, B. Gaur, L. Gauthier, P. Gauzzi, I. L. Gavrilenko, C. Gay, G. Gaycken, E. N. Gazis, P. Ge, Z. Gecse, C. N. P. Gee, D. A. A. Geerts, Ch. Geich-Gimbel, C. Gemme, M. H. Genest, S. Gentile, M. George, S. George, D. Gerbaudo, A. Gershon, H. Ghazlane, N. Ghodbane, B. Giacobbe, S. Giagu, V. Giangiobbe, P. Giannetti, F. Gianotti, B. Gibbard, S. M. Gibson, M. Gilchriese, T. P. S. Gillam, D. Gillberg, G. Gilles, D. M. Gingrich, N. Giokaris, M. P. Giordani, F. M. Giorgi, F. M. Giorgi, P. F. Giraud, D. Giugni, C. Giuliani, M. Giulini, B. K. Gjelsten, S. Gkaitatzis, I. Gkialas, E. L. Gkougkousis, L. K. Gladilin, C. Glasman, J. Glatzer, P. C. F. Glaysher, A. Glazov, G. L. Glonti, M. Goblirsch-Kolb, J. R. Goddard, J. Godlewski, S. Goldfarb, T. Golling, D. Golubkov, A. Gomes, R. Gonçalo, J. Goncalves Pinto Firmino Da Costa, L. Gonella, S. González de la Hoz, G. Gonzalez Parra, S. Gonzalez-Sevilla, L. Goossens, P. A. Gorbounov, H. A. Gordon, I. Gorelov, B. Gorini, E. Gorini, A. Gorišek, E. Gornicki, A. T. Goshaw, C. Gössling, M. I. Gostkin, M. Gouighri, D. Goujdami, A. G. Goussiou, H. M. X. Grabas, L. Graber, I. Grabowska-Bold, P. Grafström, K-J. Grahn, J. Gramling, E. Gramstad, S. Grancagnolo, V. Grassi, V. Gratchev, H. M. Gray, E. Graziani, Z. D. Greenwood, K. Gregersen, I. M. Gregor, P. Grenier, J. Griffiths, A. A. Grillo, K. Grimm, S. Grinstein, Ph. Gris, Y. V. Grishkevich, J.-F. Grivaz, J. P. Grohs, A. Grohsjean, E. Gross, J. Grosse-Knetter, G. C. Grossi, Z. J. Grout, L. Guan, J. Guenther, F. Guescini, D. Guest, O. Gueta, E. Guido, T. Guillemin, S. Guindon, U. Gul, C. Gumpert, J. Guo, S. Gupta, P. Gutierrez, N. G. Gutierrez Ortiz, C. Gutschow, N. Guttman, C. Guyot, C. Gwenlan, C. B. Gwilliam, A. Haas, C. Haber, H. K. Hadavand, N. Haddad, P. Haefner, S. Hageböck, Z. Hajduk, H. Hakobyan, M. Haleem, J. Haley, D. Hall, G. Halladjian, G. D. Hallewell, K. Hamacher, P. Hamal, K. Hamano, M. Hamer, A. Hamilton, S. Hamilton, G. N. Hamity, P. G. Hamnett, L. Han, K. Hanagaki, K. Hanawa, M. Hance, P. Hanke, R. Hanna, J. B. Hansen, J. D. Hansen, P. H. Hansen, K. Hara, A. S. Hard, T. Harenberg, F. Hariri, S. Harkusha, R. D. Harrington, P. F. Harrison, F. Hartjes, M. Hasegawa, S. Hasegawa, Y. Hasegawa, A. Hasib, S. Hassani, S. Haug, R. Hauser, L. Hauswald, M. Havranek, C. M. Hawkes, R. J. Hawkings, A. D. Hawkins, T. Hayashi, D. Hayden, C. P. Hays, J. M. Hays, H. S. Hayward, S. J. Haywood, S. J. Head, T. Heck, V. Hedberg, L. Heelan, S. Heim, T. Heim, B. Heinemann, L. Heinrich, J. Hejbal, L. Helary, M. Heller, S. Hellman, D. Hellmich, C. Helsens, J. Henderson, R. C. W. Henderson, Y. Heng, C. Hengler, A. Henrichs, A. M. Henriques Correia, S. Henrot-Versille, G. H. Herbert, Y. Hernández Jiménez, R. Herrberg-Schubert, G. Herten, R. Hertenberger, L. Hervas, G. G. Hesketh, N. P. Hessey, R. Hickling, E. Higón-Rodriguez, E. Hill, J. C. Hill, K. H. Hiller, S. J. Hillier, I. Hinchliffe, E. Hines, R. R. Hinman, M. Hirose, D. Hirschbuehl, J. Hobbs, N. Hod, M. C. Hodgkinson, P. Hodgson, A. Hoecker, M. R. Hoeferkamp, F. Hoenig, M. Hohlfeld, T. R. Holmes, T. M. Hong, L. Hooft van Huysduynen, W. H. Hopkins, Y. Horii, A. J. Horton, J-Y. Hostachy, S. Hou, A. Hoummada, J. Howard, J. Howarth, M. Hrabovsky, I. Hristova, J. Hrivnac, T. Hryn’ova, A. Hrynevich, C. Hsu, P. J. Hsu, S.-C. Hsu, D. Hu, Q. Hu, X. Hu, Y. Huang, Z. Hubacek, F. Hubaut, F. Huegging, T. B. Huffman, E. W. Hughes, G. Hughes, M. Huhtinen, T. A. Hülsing, N. Huseynov, J. Huston, J. Huth, G. Iacobucci, G. Iakovidis, I. Ibragimov, L. Iconomidou-Fayard, E. Ideal, Z. Idrissi, P. Iengo, O. Igonkina, T. Iizawa, Y. Ikegami, K. Ikematsu, M. Ikeno, Y. Ilchenko, D. Iliadis, N. Ilic, Y. Inamaru, T. Ince, P. Ioannou, M. Iodice, K. Iordanidou, V. Ippolito, A. Irles Quiles, C. Isaksson, M. Ishino, M. Ishitsuka, R. Ishmukhametov, C. Issever, S. Istin, J. M. Iturbe Ponce, R. Iuppa, J. Ivarsson, W. Iwanski, H. Iwasaki, J. M. Izen, V. Izzo, S. Jabbar, B. Jackson, M. Jackson, P. Jackson, T D Jacques, M. R. Jaekel, V. Jain, K. Jakobs, S. Jakobsen, T. Jakoubek, J. Jakubek, D. O. Jamin, D. K. Jana, E. Jansen, R. W. Jansky, J. Janssen, M. Janus, G. Jarlskog, N. Javadov, T. Javůrek, L. Jeanty, J. Jejelava, G.-Y. Jeng, D. Jennens, P. Jenni, J. Jentzsch, C. Jeske, S. Jézéquel, H. Ji, J. Jia, Y. Jiang, J. Pena, S. Jin, A. Jinaru, O. Jinnouchi, M. D. Joergensen, P. Johansson, K. A. Johns, K. Jon-And, G. Jones, R. W. L. Jones, T. J. Jones, J. Jongmanns, P. M. Jorge, K. D. Joshi, J. Jovicevic, X. Ju, C. A. Jung, P. Jussel, A. Juste Rozas, M. Kaci, A. Kaczmarska, M. Kado, H. Kagan, M. Kagan, S. J. Kahn, E. Kajomovitz, C. W. Kalderon, S. Kama, A. Kamenshchikov, N. Kanaya, M. Kaneda, S. Kaneti, V. A. Kantserov, J. Kanzaki, B. Kaplan, A. Kapliy, D. Kar, K. Karakostas, A. Karamaoun, N. Karastathis, M. J. Kareem, M. Karnevskiy, S. N. Karpov, Z. M. Karpova, K. Karthik, V. Kartvelishvili, A. N. Karyukhin, L. Kashif, R. D. Kass, A. Kastanas, Y. Kataoka, A. Katre, J. Katzy, K. Kawagoe, T. Kawamoto, G. Kawamura, S. Kazama, V. F. Kazanin, M. Y. Kazarinov, R. Keeler, R. Kehoe, M. Keil, J. S. Keller, J. J. Kempster, H. Keoshkerian, O. Kepka, B. P. Kerševan, S. Kersten, R. A. Keyes, F. Khalil-zada, H. Khandanyan, A. Khanov, A. G. Kharlamov, A. Khodinov, A. Khomich, T. J. Khoo, G. Khoriauli, V. Khovanskiy, E. Khramov, J. Khubua, H. Y. Kim, H. Kim, S. H. Kim, N. Kimura, O. M. Kind, B. T. King, M. King, R. S. B. King, S. B. King, J. Kirk, A. E. Kiryunin, T. Kishimoto, D. Kisielewska, F. Kiss, K. Kiuchi, E. Kladiva, M. H. Klein, M. Klein, U. Klein, K. Kleinknecht, P. Klimek, A. Klimentov, R. Klingenberg, J. A. Klinger, T. Klioutchnikova, P. F. Klok, E.-E. Kluge, P. Kluit, S. Kluth, E. Kneringer, E. B. F. G. Knoops, A. Knue, D. Kobayashi, T. Kobayashi, M. Kobel, M. Kocian, P. Kodys, T. Koffas, E. Koffeman, L. A. Kogan, S. Kohlmann, Z. Kohout, T. Kohriki, T. Koi, H. Kolanoski, I. Koletsou, A. A. Komar, Y. Komori, T. Kondo, N. Kondrashova, K. Köneke, A. C. König, S. König, T. Kono, R. Konoplich, N. Konstantinidis, R. Kopeliansky, S. Koperny, L. Köpke, A. K. Kopp, K. Korcyl, K. Kordas, A. Korn, A. A. Korol, I. Korolkov, E. V. Korolkova, O. Kortner, T. Kosek, S. Kortner, V. V. Kostyukhin, V. M. Kotov, A. Kotwal, A. Kourkoumeli-Charalampidi, C. Kourkoumelis, V. Kouskoura, A. Koutsman, R. Kowalewski, T. Z. Kowalski, W. Kozanecki, A. S. Kozhin, V. A. Kramarenko, G. Kramberger, D. Krasnopevtsev, M. W. Krasny, A. Krasznahorkay, J. K. Kraus, A. Kravchenko, S. Kreiss, M. Kretz, J. Kretzschmar, K. Kreutzfeldt, P. Krieger, K. Krizka, K. Kroeninger, H. Kroha, J. Kroll, J. Kroseberg, J. Krstic, U. Kruchonak, H. Krüger, N. Krumnack, Z. V. Krumshteyn, A. Kruse, M. C. Kruse, M. Kruskal, T. Kubota, H. Kucuk, S. Kuday, S. Kuehn, A. Kugel, F. Kuger, A. Kuhl, T. Kuhl, V. Kukhtin, Y. Kulchitsky, S. Kuleshov, M. Kuna, T. Kunigo, A. Kupco, H. Kurashige, Y. A. Kurochkin, R. Kurumida, V. Kus, E. S. Kuwertz, M. Kuze, J. Kvita, T. Kwan, D. Kyriazopoulos, A. La Rosa, J. L. La Rosa Navarro, L. La Rotonda, C. Lacasta, F. Lacava, J. Lacey, H. Lacker, D. Lacour, V. R. Lacuesta, E. Ladygin, R. Lafaye, B. Laforge, T. Lagouri, S. Lai, L. Lambourne, S. Lammers, C. L. Lampen, W. Lampl, E. Lançon, U. Landgraf, M. P. J. Landon, V. S. Lang, A. J. Lankford, F. Lanni, K. Lantzsch, S. Laplace, C. Lapoire, J. F. Laporte, T. Lari, F. Lasagni Manghi, M. Lassnig, P. Laurelli, W. Lavrijsen, A. T. Law, P. Laycock, O. Le Dortz, E. Le Guirriec, E. Le Menedeu, T. LeCompte, F. Ledroit-Guillon, C. A. Lee, S. C. Lee, L. Lee, G. Lefebvre, M. Lefebvre, F. Legger, C. Leggett, A. Lehan, G. Lehmann Miotto, X. Lei, W. A. Leight, A. Leisos, A. G. Leister, M. A. L. Leite, R. Leitner, D. Lellouch, B. Lemmer, K. J. C. Leney, T. Lenz, G. Lenzen, B. Lenzi, R. Leone, S. Leone, C. Leonidopoulos, S. Leontsinis, C. Leroy, C. G. Lester, M. Levchenko, J. Levêque, D. Levin, L. J. Levinson, M. Levy, A. Lewis, A. M. Leyko, M. Leyton, B. Li, B. Li, H. Li, H. L. Li, L. Li, L. Li, S. Li, Y. Li, Z. Liang, H. Liao, B. Liberti, P. Lichard, K. Lie, J. Liebal, W. Liebig, C. Limbach, A. Limosani, S. C. Lin, T. H. Lin, F. Linde, B. E. Lindquist, J. T. Linnemann, E. Lipeles, A. Lipniacka, M. Lisovyi, T. M. Liss, D. Lissauer, A. Lister, A. M. Litke, B. Liu, D. Liu, J. Liu, J. B. Liu, K. Liu, L. Liu, M. Liu, M. Liu, Y. Liu, M. Livan, A. Lleres, J. Llorente Merino, S. L. Lloyd, F. Lo Sterzo, E. Lobodzinska, P. Loch, W. S. Lockman, F. K. Loebinger, A. E. Loevschall-Jensen, A. Loginov, T. Lohse, K. Lohwasser, M. Lokajicek, B. A. Long, J. D. Long, R. E. Long, K. A. Looper, L. Lopes, D. Lopez Mateos, B. Lopez Paredes, I. Lopez Paz, J. Lorenz, N. Lorenzo Martinez, M. Losada, P. Loscutoff, P. J. Lösel, X. Lou, A. Lounis, J. Love, P. A. Love, N. Lu, H. J. Lubatti, C. Luci, A. Lucotte, F. Luehring, W. Lukas, L. Luminari, O. Lundberg, B. Lund-Jensen, M. Lungwitz, D. Lynn, R. Lysak, E. Lytken, H. Ma, L. L. Ma, G. Maccarrone, A. Macchiolo, J. Machado Mi guens, D. Macina, D. Madaffari, R. Madar, H. J. Maddocks, W. F. Mader, A. Madsen, T. Maeno, A. Maevskiy, E. Magradze, K. Mahboubi, J. Mahlstedt, S. Mahmoud, C. Maiani, C. Maidantchik, A. A. Maier, A. Maio, S. Majewski, Y. Makida, N. Makovec, B. Malaescu, Pa. Malecki, V. P. Maleev, F. Malek, U. Mallik, D. Malon, C. Malone, S. Maltezos, V. M. Malyshev, S. Malyukov, J. Mamuzic, B. Mandelli, L. Mandelli, I. Mandić, R. Mandrysch, J. Maneira, A. Manfredini, L. Manhaes de Andrade Filho, J. Manjarres Ramos, A. Mann, P. M. Manning, A. Manousakis-Katsikakis, B. Mansoulie, R. Mantifel, M. Mantoani, L. Mapelli, L. March, G. Marchiori, M. Marcisovsky, C. P. Marino, M. Marjanovic, F. Marroquim, S. P. Marsden, Z. Marshall, L. F. Marti, S. Marti-Garcia, B. Martin, T. A. Martin, V. J. Martin, B. Martin dit Latour, H. Martinez, M. Martinez, S. Martin-Haugh, A. C. Martyniuk, M. Marx, F. Marzano, A. Marzin, L. Masetti, T. Mashimo, R. Mashinistov, J. Masik, A. L. Maslennikov, I. Massa, L. Massa, N. Massol, P. Mastrandrea, A. Mastroberardino, T. Masubuchi, P. Mättig, J. Mattmann, J. Maurer, S. J. Maxfield, D. A. Maximov, R. Mazini, S. M. Mazza, L. Mazzaferro, G. Mc Goldrick, S. P. Mc Kee, A. McCarn, R. L. McCarthy, T. G. McCarthy, N. A. McCubbin, K. W. McFarlane, J. A. Mcfayden, G. Mchedlidze, S. J. McMahon, R. A. McPherson, J. Mechnich, M. Medinnis, S. Meehan, S. Mehlhase, A. Mehta, K. Meier, C. Meineck, B. Meirose, C. Melachrinos, B. R. Mellado Garcia, F. Meloni, A. Mengarelli, S. Menke, E. Meoni, K. M. Mercurio, S. Mergelmeyer, N. Meric, P. Mermod, L. Merola, C. Meroni, F. S. Merritt, H. Merritt, A. Messina, J. Metcalfe, A. S. Mete, C. Meyer, C. Meyer, J-P. Meyer, J. Meyer, R. P. Middleton, S. Migas, S. Miglioranzi, L. Mijović, G. Mikenberg, M. Mikestikova, M. Mikuž, A. Milic, D. W. Miller, C. Mills, A. Milov, D. A. Milstead, A. A. Minaenko, Y. Minami, I. A. Minashvili, A. I. Mincer, B. Mindur, M. Mineev, Y. Ming, L. M. Mir, G. Mirabelli, T. Mitani, J. Mitrevski, V. A. Mitsou, A. Miucci, P. S. Miyagawa, J. U. Mjörnmark, T. Moa, K. Mochizuki, S. Mohapatra, W. Mohr, S. Molander, R. Moles-Valls, K. Mönig, C. Monini, J. Monk, E. Monnier, J. Montejo Berlingen, F. Monticelli, S. Monzani, R. W. Moore, N. Morange, D. Moreno, M. Moreno Llácer, P. Morettini, M. Morgenstern, M. Morii, V. Morisbak, S. Moritz, A. K. Morley, G. Mornacchi, J. D. Morris, A. Morton, L. Morvaj, H. G. Moser, M. Mosidze, J. Moss, K. Motohashi, R. Mount, E. Mountricha, S. V. Mouraviev, E. J. W. Moyse, S. Muanza, R. D. Mudd, F. Mueller, J. Mueller, K. Mueller, R. S. P. Mueller, T. Mueller, D. Muenstermann, P. Mullen, Y. Munwes, J. A. Murillo Quijada, W. J. Murray, H. Musheghyan, E. Musto, A. G. Myagkov, M. Myska, O. Nackenhorst, J. Nadal, K. Nagai, R. Nagai, Y. Nagai, K. Nagano, A. Nagarkar, Y. Nagasaka, K. Nagata, M. Nagel, E. Nagy, A. M. Nairz, Y. Nakahama, K. Nakamura, T. Nakamura, I. Nakano, H. Namasivayam, G. Nanava, R. F. Naranjo Garcia, R. Narayan, T. Nattermann, T. Naumann, G. Navarro, R. Nayyar, H. A. Neal, P. Yu. Nechaeva, T. J. Neep, P. D. Nef, A. Negri, M. Negrini, S. Nektarijevic, C. Nellist, A. Nelson, S. Nemecek, P. Nemethy, A. A. Nepomuceno, M. Nessi, M. S. Neubauer, M. Neumann, R. M. Neves, P. Nevski, P. R. Newman, D. H. Nguyen, R. B. Nickerson, R. Nicolaidou, B. Nicquevert, J. Nielsen, N. Nikiforou, A. Nikiforov, V. Nikolaenko, I. Nikolic-Audit, K. Nikolopoulos, P. Nilsson, Y. Ninomiya, A. Nisati, R. Nisius, T. Nobe, M. Nomachi, I. Nomidis, S. Norberg, M. Nordberg, O. Novgorodova, S. Nowak, M. Nozaki, L. Nozka, K. Ntekas, G. Nunes Hanninger, T. Nunnemann, E. Nurse, F. Nuti, B. J. O’Brien, F. O’grady, D. C. O’Neil, V. O’Shea, F. G. Oakham, H. Oberlack, T. Obermann, J. Ocariz, A. Ochi, I. Ochoa, S. Oda, S. Odaka, H. Ogren, A. Oh, S. H. Oh, C. C. Ohm, H. Ohman, H. Oide, W. Okamura, H. Okawa, Y. Okumura, T. Okuyama, A. Olariu, A. G. Olchevski, S. A. Olivares Pino, D. Oliveira Damazio, E. Oliver Garcia, A. Olszewski, J. Olszowska, A. Onofre, P. U. E. Onyisi, C. J. Oram, M. J. Oreglia, Y. Oren, D. Orestano, N. Orlando, C. Oropeza Barrera, R. S. Orr, B. Osculati, R. Ospanov, G. Otero y Garzon, H. Otono, M. Ouchrif, E. A. Ouellette, F. Ould-Saada, A. Ouraou, K. P. Oussoren, Q. Ouyang, A. Ovcharova, M. Owen, R. E. Owen, V. E. Ozcan, N. Ozturk, K. Pachal, A. Pacheco Pages, C. Padilla Aranda, M. Pagáčová, S. Pagan Griso, E. Paganis, C. Pahl, F. Paige, P. Pais, K. Pajchel, G. Palacino, S. Palestini, M. Palka, D. Pallin, A. Palma, Y. B. Pan, E. Panagiotopoulou, C. E. Pandini, J. G. Panduro Vazquez, P. Pani, N. Panikashvili, S. Panitkin, L. Paolozzi, Th. D. Papadopoulou, K. Papageorgiou, A. Paramonov, D. Paredes Hernandez, M. A. Parker, K. A. Parker, F. Parodi, J. A. Parsons, U. Parzefall, E. Pasqualucci, S. Passaggio, F. Pastore, Fr. Pastore, G. Pásztor, S. Pataraia, N. D. Patel, J. R. Pater, T. Pauly, J. Pearce, L. E. Pedersen, M. Pedersen, S. Pedraza Lopez, R. Pedro, S. V. Peleganchuk, D. Pelikan, H. Peng, B. Penning, J. Penwell, D. V. Perepelitsa, E. Perez Codina, M. T. Pérez García-Estañ, L. Perini, H. Pernegger, S. Perrella, R. Peschke, V. D. Peshekhonov, K. Peters, R. F. Y. Peters, B. A. Petersen, T. C. Petersen, E. Petit, A. Petridis, C. Petridou, E. Petrolo, F. Petrucci, N. E. Pettersson, R. Pezoa, P. W. Phillips, G. Piacquadio, E. Pianori, A. Picazio, E. Piccaro, M. Piccinini, M. A. Pickering, R. Piegaia, D. T. Pignotti, J. E. Pilcher, A. D. Pilkington, J. Pina, M. Pinamonti, J. L. Pinfold, A. Pingel, B. Pinto, S. Pires, M. Pitt, C. Pizio, L. Plazak, M.-A. Pleier, V. Pleskot, E. Plotnikova, P. Plucinski, D. Pluth, R. Poettgen, L. Poggioli, D. Pohl, G. Polesello, A. Policicchio, R. Polifka, A. Polini, C. S. Pollard, V. Polychronakos, K. Pommès, L. Pontecorvo, B. G. Pope, G. A. Popeneciu, D. S. Popovic, A. Poppleton, S. Pospisil, K. Potamianos, I. N. Potrap, C. J. Potter, C. T. Potter, G. Poulard, J. Poveda, V. Pozdnyakov, P. Pralavorio, A. Pranko, S. Prasad, S. Prell, D. Price, J. Price, L. E. Price, M. Primavera, S. Price, M. Proissl, K. Prokofiev, F. Prokoshin, E. Protopapadaki, S. Protopopescu, J. Proudfoot, M. Przybycien, E. Ptacek, D. Puddu, E. Pueschel, D. Puldon, M. Purohit, P. Puzo, J. Qian, G. Qin, Y. Qin, A. Quadt, D. R. Quarrie, W. B. Quayle, M. Queitsch-Maitland, D. Quilty, A. Qureshi, V. Radeka, V. Radescu, S. K. Radhakrishnan, P. Radloff, P. Rados, F. Ragusa, G. Rahal, S. Rajagopalan, M. Rammensee, C. Rangel-Smith, F. Rauscher, S. Rave, T. C. Rave, T. Ravenscroft, M. Raymond, A. L. Read, N. P. Readioff, D. M. Rebuzzi, A. Redelbach, G. Redlinger, R. Reece, K. Reeves, L. Rehnisch, H. Reisin, M. Relich, C. Rembser, H. Ren, A. Renaud, M. Rescigno, S. Resconi, O. L. Rezanova, P. Reznicek, R. Rezvani, R. Richter, E. Richter-Was, M. Ridel, P. Rieck, C. J. Riegel, J. Rieger, M. Rijssenbeek, A. Rimoldi, L. Rinaldi, A. W. Riotto, E. Ritsch, I. Riu, F. Rizatdinova, E. Rizvi, S. H. Robertson, A. Robichaud-Veronneau, D. Robinson, J. E. M. Robinson, A. Robson, C. Roda, L. Rodrigues, S. Roe, O. Røhne, S. Rolli, A. Romaniouk, M. Romano, S. M. Romano Saez, E. Romero Adam, N. Rompotis, M. Ronzani, L. Roos, E. Ros, S. Rosati, K. Rosbach, P. Rose, P. L. Rosendahl, O. Rosenthal, V. Rossetti, E. Rossi, L. P. Rossi, R. Rosten, M. Rotaru, I. Roth, J. Rothberg, D. Rousseau, C. R. Royon, A. Rozanov, Y. Rozen, X. Ruan, F. Rubbo, I. Rubinskiy, V. I. Rud, C. Rudolph, M. S. Rudolph, F. Rühr, A. Ruiz-Martinez, Z. Rurikova, N. A. Rusakovich, A. Ruschke, H. L. Russell, J. P. Rutherfoord, N. Ruthmann, Y. F. Ryabov, M. Rybar, G. Rybkin, N. C. Ryder, A. F. Saavedra, G. Sabato, S. Sacerdoti, A. Saddique, H.F-W. Sadrozinski, R. Sadykov, F. Safai Tehrani, M. Saimpert, H. Sakamoto, Y. Sakurai, G. Salamanna, A. Salamon, M. Saleem, D. Salek, P. H. Sales De Bruin, D. Salihagic, A. Salnikov, J. Salt, D. Salvatore, F. Salvatore, A. Salvucci, A. Salzburger, D. Sampsonidis, A. Sanchez, J. Sánchez, V. Sanchez Martinez, H. Sandaker, R. L. Sandbach, H. G. Sander, M. P. Sanders, M. Sandhoff, C. Sandoval, R. Sandstroem, D. P. C. Sankey, A. Sansoni, C. Santoni, R. Santonico, H. Santos, I. Santoyo Castillo, K. Sapp, A. Sapronov, J. G. Saraiva, B. Sarrazin, O. Sasaki, Y. Sasaki, K. Sato, G. Sauvage, E. Sauvan, G. Savage, P. Savard, C. Sawyer, L. Sawyer, D. H. Saxon, J. Saxon, C. Sbarra, A. Sbrizzi, T. Scanlon, D. A. Scannicchio, M. Scarcella, V. Scarfone, J. Schaarschmidt, P. Schacht, D. Schaefer, R. Schaefer, J. Schaeffer, S. Schaepe, S. Schaetzel, U. Schäfer, A. C. Schaffer, D. Schaile, R. D. Schamberger, V. Scharf, V. A. Schegelsky, D. Scheirich, M. Schernau, C. Schiavi, C. Schillo, M. Schioppa, S. Schlenker, E. Schmidt, K. Schmieden, C. Schmitt, S. Schmitt, B. Schneider, Y. J. Schnellbach, U. Schnoor, L. Schoeffel, A. Schoening, B. D. Schoenrock, A. L. S. Schorlemmer, M. Schott, D. Schouten, J. Schovancova, S. Schramm, M. Schreyer, C. Schroeder, N. Schuh, M. J. Schultens, H.-C. Schultz-Coulon, H. Schulz, M. Schumacher, B. A. Schumm, Ph. Schune, C. Schwanenberger, A. Schwartzman, T. A. Schwarz, Ph. Schwegler, Ph. Schwemling, R. Schwienhorst, J. Schwindling, T. Schwindt, M. Schwoerer, F. G. Sciacca, E. Scifo, G. Sciolla, F. Scuri, F. Scutti, J. Searcy, G. Sedov, E. Sedykh, P. Seema, S. C. Seidel, A. Seiden, F. Seifert, J. M. Seixas, G. Sekhniaidze, S. J. Sekula, K. E. Selbach, D. M. Seliverstov, N. Semprini-Cesari, C. Serfon, L. Serin, L. Serkin, T. Serre, R. Seuster, H. Severini, T. Sfiligoj, F. Sforza, A. Sfyrla, E. Shabalina, M. Shamim, L. Y. Shan, R. Shang, J. T. Shank, M. Shapiro, P. B. Shatalov, K. Shaw, A. Shcherbakova, C. Y. Shehu, P. Sherwood, L. Shi, S. Shimizu, C. O. Shimmin, M. Shimojima, M. Shiyakova, A. Shmeleva, D. Shoaleh Saadi, M. J. Shochet, S. Shojaii, S. Shrestha, E. Shulga, M. A. Shupe, S. Shushkevich, P. Sicho, O. Sidiropoulou, D. Sidorov, A. Sidoti, F. Siegert, Dj. Sijacki, J. Silva, Y. Silver, D. Silverstein, S. B. Silverstein, V. Simak, O. Simard, Lj. Simic, S. Simion, E. Simioni, B. Simmons, D. Simon, R. Simoniello, P. Sinervo, N. B. Sinev, G. Siragusa, A. Sircar, A. N. Sisakyan, S. Yu. Sivoklokov, J. Sjölin, T. B. Sjursen, M. B. Skinner, H. P. Skottowe, P. Skubic, M. Slater, T. Slavicek, M. Slawinksa, K. Sliwa, V. Smakhtin, B. H. Smart, L. Smestad, S. Yu. Smirnov, Y. Smirnov, L. N. Smirnova, O. Smirnova, K. M. Smith, M. N. K. Smith, M. Smizanska, K. Smolek, A. A. Snesarev, G. Snidero, S. Snyder, R. Sobie, F. Socher, A. Soffer, D. A. Soh, C. A. Solans, M. Solar, J. Solc, E. Yu. Soldatov, U. Soldevila, A. A. Solodkov, A. Soloshenko, O. V. Solovyanov, V. Solovyev, P. Sommer, H. Y. Song, N. Soni, A. Sood, A. Sopczak, B. Sopko, V. Sopko, V. Sorin, D. Sosa, M. Sosebee, C. L. Sotiropoulou, R. Soualah, P. Soueid, A. M. Soukharev, D. South, S. Spagnolo, F. Spanò, W. R. Spearman, F. Spettel, R. Spighi, G. Spigo, L. A. Spiller, M. Spousta, T. Spreitzer, R. D. St. Denis, S. Staerz, J. Stahlman, R. Stamen, S. Stamm, E. Stanecka, C. Stanescu, M. Stanescu-Bellu, M. M. Stanitzki, S. Stapnes, E. A. Starchenko, J. Stark, P. Staroba, P. Starovoitov, R. Staszewski, P. Stavina, P. Steinberg, B. Stelzer, H. J. Stelzer, O. Stelzer-Chilton, H. Stenzel, S. Stern, G. A. Stewart, J. A. Stillings, M. C. Stockton, M. Stoebe, G. Stoicea, P. Stolte, S. Stonjek, A. R. Stradling, A. Straessner, M. E. Stramaglia, J. Strandberg, S. Strandberg, A. Strandlie, E. Strauss, M. Strauss, P. Strizenec, R. Ströhmer, D. M. Strom, R. Stroynowski, A. Strubig, S. A. Stucci, B. Stugu, N. A. Styles, D. Su, J. Su, R. Subramaniam, A. Succurro, Y. Sugaya, C. Suhr, M. Suk, V. V. Sulin, S. Sultansoy, T. Sumida, S. Sun, X. Sun, J. E. Sundermann, K. Suruliz, G. Susinno, M. R. Sutton, Y. Suzuki, M. Svatos, S. Swedish, M. Swiatlowski, I. Sykora, T. Sykora, D. Ta, C. Taccini, K. Tackmann, J. Taenzer, A. Taffard, R. Tafirout, N. Taiblum, H. Takai, R. Takashima, H. Takeda, T. Takeshita, Y. Takubo, M. Talby, A. A. Talyshev, J. Y. C. Tam, K. G. Tan, J. Tanaka, R. Tanaka, S. Tanaka, S. Tanaka, A. J. Tanasijczuk, B. B. Tannenwald, N. Tannoury, S. Tapprogge, S. Tarem, F. Tarrade, G. F. Tartarelli, P. Tas, M. Tasevsky, T. Tashiro, E. Tassi, A. Tavares Delgado, Y. Tayalati, F. E. Taylor, G. N. Taylor, W. Taylor, F. A. Teischinger, M. Teixeira Dias Castanheira, P. Teixeira-Dias, K. K. Temming, H. Ten Kate, P. K. Teng, J. J. Teoh, F. Tepel, S. Terada, K. Terashi, J. Terron, S. Terzo, M. Testa, R. J. Teuscher, J. Therhaag, T. Theveneaux-Pelzer, J. P. Thomas, J. Thomas-Wilsker, E. N. Thompson, P. D. Thompson, R. J. Thompson, A. S. Thompson, L. A. Thomsen, E. Thomson, M. Thomson, W. M. Thong, R. P. Thun, F. Tian, M. J. Tibbetts, R. E. Ticse Torres, V. O. Tikhomirov, Yu. A. Tikhonov, S. Timoshenko, E. Tiouchichine, P. Tipton, S. Tisserant, T. Todorov, S. Todorova-Nova, J. Tojo, S. Tokár, K. Tokushuku, K. Tollefson, E. Tolley, L. Tomlinson, M. Tomoto, L. Tompkins, K. Toms, N. D. Topilin, E. Torrence, H. Torres, E. Torró Pastor, J. Toth, F. Touchard, D. R. Tovey, H. L. Tran, T. Trefzger, L. Tremblet, A. Tricoli, I. M. Trigger, S. Trincaz-Duvoid, M. F. Tripiana, W. Trischuk, B. Trocmé, C. Troncon, M. Trottier-McDonald, M. Trovatelli, P. True, M. Trzebinski, A. Trzupek, C. Tsarouchas, J.C-L. Tseng, P. V. Tsiareshka, D. Tsionou, G. Tsipolitis, N. Tsirintanis, S. Tsiskaridze, V. Tsiskaridze, E. G. Tskhadadze, I. I. Tsukerman, V. Tsulaia, S. Tsuno, D. Tsybychev, A. Tudorache, V. Tudorache, A. N. Tuna, S. A. Tupputi, S. Turchikhin, D. Turecek, R. Turra, A. J. Turvey, P. M. Tuts, A. Tykhonov, M. Tylmad, M. Tyndel, I. Ueda, R. Ueno, M. Ughetto, M. Ugland, M. Uhlenbrock, F. Ukegawa, G. Unal, A. Undrus, G. Unel, F. C. Ungaro, Y. Unno, C. Unverdorben, J. Urban, P. Urquijo, P. Urrejola, G. Usai, A. Usanova, L. Vacavant, V. Vacek, B. Vachon, N. Valencic, S. Valentinetti, A. Valero, L. Valery, S. Valkar, E. Valladolid Gallego, S. Vallecorsa, J. A. Valls Ferrer, W. Van Den Wollenberg, P. C. Van Der Deijl, R. van der Geer, H. van der Graaf, R. Van Der Leeuw, N. van Eldik, P. van Gemmeren, J. Van Nieuwkoop, I. van Vulpen, M. C. van Woerden, M. Vanadia, W. Vandelli, R. Vanguri, A. Vaniachine, F. Vannucci, G. Vardanyan, R. Vari, E. W. Varnes, T. Varol, D. Varouchas, A. Vartapetian, K. E. Varvell, F. Vazeille, T. Vazquez Schroeder, J. Veatch, F. Veloso, T. Velz, S. Veneziano, A. Ventura, D. Ventura, M. Venturi, N. Venturi, A. Venturini, V. Vercesi, M. Verducci, W. Verkerke, J. C. Vermeulen, A. Vest, M. C. Vetterli, O. Viazlo, I. Vichou, T. Vickey, O. E. Vickey Boeriu, G. H. A. Viehhauser, S. Viel, R. Vigne, M. Villa, M. Villaplana Perez, E. Vilucchi, M. G. Vincter, V. B. Vinogradov, J. Virzi, I. Vivarelli, F. Vives Vaque, S. Vlachos, D. Vladoiu, M. Vlasak, M. Vogel, P. Vokac, G. Volpi, M. Volpi, H. von der Schmitt, H. von Radziewski, E. von Toerne, V. Vorobel, K. Vorobev, M. Vos, R. Voss, J. H. Vossebeld, N. Vranjes, M. Vranjes Milosavljevic, V. Vrba, M. Vreeswijk, R. Vuillermet, I. Vukotic, Z. Vykydal, P. Wagner, W. Wagner, H. Wahlberg, S. Wahrmund, J. Wakabayashi, J. Walder, R. Walker, W. Walkowiak, C. Wang, F. Wang, H. Wang, H. Wang, J. Wang, J. Wang, K. Wang, R. Wang, S. M. Wang, T. Wang, X. Wang, C. Wanotayaroj, A. Warburton, C. P. Ward, D. R. Wardrope, M. Warsinsky, A. Washbrook, C. Wasicki, P. M. Watkins, A. T. Watson, I. J. Watson, M. F. Watson, G. Watts, S. Watts, B. M. Waugh, S. Webb, M. S. Weber, S. W. Weber, J. S. Webster, A. R. Weidberg, B. Weinert, J. Weingarten, C. Weiser, H. Weits, P. S. Wells, T. Wenaus, D. Wendland, T. Wengler, S. Wenig, N. Wermes, M. Werner, P. Werner, M. Wessels, J. Wetter, K. Whalen, A. M. Wharton, A. White, M. J. White, R. White, S. White, D. Whiteson, D. Wicke, F. J. Wickens, W. Wiedenmann, M. Wielers, P. Wienemann, C. Wiglesworth, L. A. M. Wiik-Fuchs, A. Wildauer, H. G. Wilkens, H. H. Williams, S. Williams, C. Willis, S. Willocq, A. Wilson, J. A. Wilson, I. Wingerter-Seez, F. Winklmeier, B. T. Winter, M. Wittgen, J. Wittkowski, S. J. Wollstadt, M. W. Wolter, H. Wolters, B. K. Wosiek, J. Wotschack, M. J. Woudstra, K. W. Wozniak, M. Wu, S. L. Wu, X. Wu, Y. Wu, T. R. Wyatt, B. M. Wynne, S. Xella, D. Xu, L. Xu, B. Yabsley, S. Yacoob, R. Yakabe, M. Yamada, Y. Yamaguchi, A. Yamamoto, S. Yamamoto, T. Yamanaka, K. Yamauchi, Y. Yamazaki, Z. Yan, H. Yang, H. Yang, Y. Yang, S. Yanush, L. Yao, W-M. Yao, Y. Yasu, E. Yatsenko, K. H. Yau Wong, J. Ye, S. Ye, I. Yeletskikh, A. L. Yen, E. Yildirim, K. Yorita, R. Yoshida, K. Yoshihara, C. Young, C. J. S. Young, S. Youssef, D. R. Yu, J. Yu, J. M. Yu, J. Yu, L. Yuan, A. Yurkewicz, I. Yusuff, B. Zabinski, R. Zaidan, A. M. Zaitsev, A. Zaman, S. Zambito, L. Zanello, D. Zanzi, C. Zeitnitz, M. Zeman, A. Zemla, K. Zengel, O. Zenin, T. Ženiš, D. Zerwas, D. Zhang, F. Zhang, J. Zhang, L. Zhang, R. Zhang, X. Zhang, Z. Zhang, X. Zhao, Y. Zhao, Z. Zhao, A. Zhemchugov, J. Zhong, B. Zhou, C. Zhou, L. Zhou, L. Zhou, N. Zhou, C. G. Zhu, H. Zhu, J. Zhu, Y. Zhu, X. Zhuang, K. Zhukov, A. Zibell, D. Zieminska, N. I. Zimine, C. Zimmermann, R. Zimmermann, S. Zimmermann, Z. Zinonos, M. Zinser, M. Ziolkowski, L. Živković, G. Zobernig, A. Zoccoli, M. zur Nedden, G. Zurzolo, L. Zwalinski, ATLAS Collaboration

**Affiliations:** Department of Physics, University of Adelaide, Adelaide, Australia; Physics Department, SUNY Albany, Albany, NY USA; Department of Physics, University of Alberta, Edmonton, AB Canada; Department of Physics, Ankara University, Ankara, Turkey; Istanbul Aydin University, Istanbul, Turkey; Division of Physics, TOBB University of Economics and Technology, Ankara, Turkey; LAPP, CNRS/IN2P3 and Université Savoie Mont Blanc, Annecy-le-Vieux, France; High Energy Physics Division, Argonne National Laboratory, Argonne, IL USA; Department of Physics, University of Arizona, Tucson, AZ USA; Department of Physics, The University of Texas at Arlington, Arlington, TX USA; Physics Department, University of Athens, Athens, Greece; Physics Department, National Technical University of Athens, Zografou, Greece; Institute of Physics, Azerbaijan Academy of Sciences, Baku, Azerbaijan; Institut de Física d’Altes Energies and Departament de Física de la Universitat Autònoma de Barcelona, Barcelona, Spain; Institute of Physics, University of Belgrade, Belgrade, Serbia; Department for Physics and Technology, University of Bergen, Bergen, Norway; Physics Division, Lawrence Berkeley National Laboratory and University of California, Berkeley, CA USA; Department of Physics, Humboldt University, Berlin, Germany; Albert Einstein Center for Fundamental Physics and Laboratory for High Energy Physics, University of Bern, Bern, Switzerland; School of Physics and Astronomy, University of Birmingham, Birmingham, UK; Department of Physics, Bogazici University, Istanbul, Turkey; Department of Physics, Dogus University, Istanbul, Turkey; Department of Physics Engineering, Gaziantep University, Gaziantep, Turkey; INFN Sezione di Bologna, Bologna, Italy; Dipartimento di Fisica e Astronomia, Università di Bologna, Bologna, Italy; Physikalisches Institut, University of Bonn, Bonn, Germany; Department of Physics, Boston University, Boston, MA USA; Department of Physics, Brandeis University, Waltham, MA USA; Universidade Federal do Rio De Janeiro COPPE/EE/IF, Rio de Janeiro, Brazil; Electrical Circuits Department, Federal University of Juiz de Fora (UFJF), Juiz de Fora, Brazil; Federal University of Sao Joao del Rei (UFSJ), Sao Joao del Rei, Brazil; Instituto de Fisica, Universidade de Sao Paulo, São Paulo, Brazil; Physics Department, Brookhaven National Laboratory, Upton, NY USA; National Institute of Physics and Nuclear Engineering, Bucharest, Romania; Physics Department, National Institute for Research and Development of Isotopic and Molecular Technologies, Cluj Napoca, Romania; University Politehnica Bucharest, Bucharest, Romania; West University in Timisoara, Timisoara, Romania; Departamento de Física, Universidad de Buenos Aires, Buenos Aires, Argentina; Cavendish Laboratory, University of Cambridge, Cambridge, UK; Department of Physics, Carleton University, Ottawa, ON Canada; CERN, Geneva, Switzerland; Enrico Fermi Institute, University of Chicago, Chicago, IL USA; Departamento de Física, Pontificia Universidad Católica de Chile, Santiago, Chile; Departamento de Física, Universidad Técnica Federico Santa María, Valparaiso, Chile; Institute of High Energy Physics, Chinese Academy of Sciences, Beijing, China; Department of Modern Physics, University of Science and Technology of China, Anhui, China; Department of Physics, Nanjing University, Jiangsu, China; School of Physics, Shandong University, Shandong, China; Department of Physics and Astronomy, Shanghai Key Laboratory for Particle Physics and Cosmology, Shanghai Jiao Tong University, Shanghai, China; Physics Department, Tsinghua University, 100084 Beijing, China; Laboratoire de Physique Corpusculaire, Clermont Université and Université Blaise Pascal and CNRS/IN2P3, Clermont-Ferrand, France; Nevis Laboratory, Columbia University, Irvington, NY USA; Niels Bohr Institute, University of Copenhagen, Copenhagen, Denmark; INFN Gruppo Collegato di Cosenza, Laboratori Nazionali di Frascati, Frascati, Italy; Dipartimento di Fisica, Università della Calabria, Rende, Italy; Faculty of Physics and Applied Computer Science, AGH University of Science and Technology, Kraków, Poland; Marian Smoluchowski Institute of Physics, Jagiellonian University, Kraków, Poland; Institute of Nuclear Physics, Polish Academy of Sciences, Kraków, Poland; Physics Department, Southern Methodist University, Dallas, TX USA; Physics Department, University of Texas at Dallas, Richardson, TX USA; DESY, Hamburg and Zeuthen, Germany; Institut für Experimentelle Physik IV, Technische Universität Dortmund, Dortmund, Germany; Institut für Kern- und Teilchenphysik, Technische Universität Dresden, Dresden, Germany; Department of Physics, Duke University, Durham, NC USA; SUPA-School of Physics and Astronomy, University of Edinburgh, Edinburgh, UK; INFN Laboratori Nazionali di Frascati, Frascati, Italy; Fakultät für Mathematik und Physik, Albert-Ludwigs-Universität, Freiburg, Germany; Section de Physique, Université de Genève, Geneva, Switzerland; INFN Sezione di Genova, Genova, Italy; Dipartimento di Fisica, Università di Genova, Genoa, Italy; E. Andronikashvili Institute of Physics, Iv. Javakhishvili Tbilisi State University, Tbilisi, Georgia; High Energy Physics Institute, Tbilisi State University, Tbilisi, Georgia; II Physikalisches Institut, Justus-Liebig-Universität Giessen, Giessen, Germany; SUPA-School of Physics and Astronomy, University of Glasgow, Glasgow, UK; II Physikalisches Institut, Georg-August-Universität, Göttingen, Germany; Laboratoire de Physique Subatomique et de Cosmologie, Université Grenoble-Alpes, CNRS/IN2P3, Grenoble, France; Department of Physics, Hampton University, Hampton, VA USA; Laboratory for Particle Physics and Cosmology, Harvard University, Cambridge, MA USA; Kirchhoff-Institut für Physik, Ruprecht-Karls-Universität Heidelberg, Heidelberg, Germany; Physikalisches Institut, Ruprecht-Karls-Universität Heidelberg, Heidelberg, Germany; ZITI Institut für technische Informatik, Ruprecht-Karls-Universität Heidelberg, Mannheim, Germany; Faculty of Applied Information Science, Hiroshima Institute of Technology, Hiroshima, Japan; Department of Physics, The Chinese University of Hong Kong, Shatin, NT, Hong Kong; Department of Physics, The University of Hong Kong, Pok Fu Lam, Hong Kong; Department of Physics, The Hong Kong University of Science and Technology, Clear Water Bay, Kowloon, Hong Kong, China; Department of Physics, Indiana University, Bloomington, IN USA; Institut für Astro- und Teilchenphysik, Leopold-Franzens-Universität, Innsbruck, Austria; University of Iowa, Iowa City, IA USA; Department of Physics and Astronomy, Iowa State University, Ames, IA USA; Joint Institute for Nuclear Research, JINR Dubna, Dubna, Russia; KEK, High Energy Accelerator Research Organization, Tsukuba, Japan; Graduate School of Science, Kobe University, Kobe, Japan; Faculty of Science, Kyoto University, Kyoto, Japan; Kyoto University of Education, Kyoto, Japan; Department of Physics, Kyushu University, Fukuoka, Japan; Instituto de Física La Plata, Universidad Nacional de La Plata and CONICET, La Plata, Argentina; Physics Department, Lancaster University, Lancaster, UK; INFN Sezione di Lecce, Lecce, Italy; Dipartimento di Matematica e Fisica, Università del Salento, Lecce, Italy; Oliver Lodge Laboratory, University of Liverpool, Liverpool, UK; Department of Physics, Jožef Stefan Institute and University of Ljubljana, Ljubljana, Slovenia; School of Physics and Astronomy, Queen Mary University of London, London, UK; Department of Physics, Royal Holloway University of London, Surrey, UK; Department of Physics and Astronomy, University College London, London, UK; Louisiana Tech University, Ruston, LA USA; Laboratoire de Physique Nucléaire et de Hautes Energies, UPMC and Université Paris-Diderot and CNRS/IN2P3, Paris, France; Fysiska institutionen, Lunds universitet, Lund, Sweden; Departamento de Fisica Teorica C-15, Universidad Autonoma de Madrid, Madrid, Spain; Institut für Physik, Universität Mainz, Mainz, Germany; School of Physics and Astronomy, University of Manchester, Manchester, UK; CPPM, Aix-Marseille Université and CNRS/IN2P3, Marseille, France; Department of Physics, University of Massachusetts, Amherst, MA USA; Department of Physics, McGill University, Montreal, QC Canada; School of Physics, University of Melbourne, Melbourne, VIC Australia; Department of Physics, The University of Michigan, Ann Arbor, MI USA; Department of Physics and Astronomy, Michigan State University, East Lansing, MI USA; INFN Sezione di Milano, Milan, Italy; Dipartimento di Fisica, Università di Milano, Milan, Italy; B.I. Stepanov Institute of Physics, National Academy of Sciences of Belarus, Minsk, Republic of Belarus; National Scientific and Educational Centre for Particle and High Energy Physics, Minsk, Republic of Belarus; Department of Physics, Massachusetts Institute of Technology, Cambridge, MA USA; Group of Particle Physics, University of Montreal, Montreal, QC Canada; P.N. Lebedev Institute of Physics, Academy of Sciences, Moscow, Russia; Institute for Theoretical and Experimental Physics (ITEP), Moscow, Russia; National Research Nuclear University MEPhI, Moscow, Russia; D.V. Skobeltsyn Institute of Nuclear Physics, M.V. Lomonosov Moscow State University, Moscow, Russia; Fakultät für Physik, Ludwig-Maximilians-Universität München, Munich, Germany; Max-Planck-Institut für Physik (Werner-Heisenberg-Institut), Munich, Germany; Nagasaki Institute of Applied Science, Nagasaki, Japan; Graduate School of Science and Kobayashi-Maskawa Institute, Nagoya University, Nagoya, Japan; INFN Sezione di Napoli, Naples, Italy; Dipartimento di Fisica, Università di Napoli, Naples, Italy; Department of Physics and Astronomy, University of New Mexico, Albuquerque, NM USA; Institute for Mathematics, Astrophysics and Particle Physics, Radboud University Nijmegen/Nikhef, Nijmegen, The Netherlands; Nikhef National Institute for Subatomic Physics and University of Amsterdam, Amsterdam, The Netherlands; Department of Physics, Northern Illinois University, De Kalb, IL USA; Budker Institute of Nuclear Physics, SB RAS, Novosibirsk, Russia; Department of Physics, New York University, New York, NY USA; Ohio State University, Columbus, OH USA; Faculty of Science, Okayama University, Okayama, Japan; Homer L. Dodge Department of Physics and Astronomy, University of Oklahoma, Norman, OK USA; Department of Physics, Oklahoma State University, Stillwater, OK USA; Palacký University, RCPTM, Olomouc, Czech Republic; Center for High Energy Physics, University of Oregon, Eugene, OR USA; LAL, Université Paris-Sud and CNRS/IN2P3, Orsay, France; Graduate School of Science, Osaka University, Osaka, Japan; Department of Physics, University of Oslo, Oslo, Norway; Department of Physics, Oxford University, Oxford, UK; INFN Sezione di Pavia, Pavia, Italy; Dipartimento di Fisica, Università di Pavia, Pavia, Italy; Department of Physics, University of Pennsylvania, Philadelphia, PA USA; Petersburg Nuclear Physics Institute, Gatchina, Russia; INFN Sezione di Pisa, Pisa, Italy; Dipartimento di Fisica E. Fermi, Università di Pisa, Pisa, Italy; Department of Physics and Astronomy, University of Pittsburgh, Pittsburgh, PA USA; Laboratorio de Instrumentacao e Fisica Experimental de Particulas-LIP, Lisbon, Portugal; Faculdade de Ciências, Universidade de Lisboa, Lisbon, Portugal; Department of Physics, University of Coimbra, Coimbra, Portugal; Centro de Física Nuclear da Universidade de Lisboa, Lisbon, Portugal; Departamento de Fisica, Universidade do Minho, Braga, Portugal; Departamento de Fisica Teorica y del Cosmos and CAFPE, Universidad de Granada, Granada, Spain; Dep Fisica and CEFITEC of Faculdade de Ciencias e Tecnologia, Universidade Nova de Lisboa, Caparica, Portugal; Institute of Physics, Academy of Sciences of the Czech Republic, Prague, Czech Republic; Czech Technical University in Prague, Prague, Czech Republic; Faculty of Mathematics and Physics, Charles University in Prague, Prague, Czech Republic; State Research Center Institute for High Energy Physics, Protvino, Russia; Particle Physics Department, Rutherford Appleton Laboratory, Didcot, UK; Ritsumeikan University, Kusatsu, Shiga Japan; INFN Sezione di Roma, Rome, Italy; Dipartimento di Fisica, Sapienza Università di Roma, Rome, Italy; INFN Sezione di Roma Tor Vergata, Rome, Italy; Dipartimento di Fisica, Università di Roma Tor Vergata, Rome, Italy; INFN Sezione di Roma Tre, Rome, Italy; Dipartimento di Matematica e Fisica, Università Roma Tre, Rome, Italy; Faculté des Sciences Ain Chock, Réseau Universitaire de Physique des Hautes Energies-Université Hassan II, Casablanca, Morocco; Centre National de l’Energie des Sciences Techniques Nucleaires, Rabat, Morocco; Faculté des Sciences Semlalia, Université Cadi Ayyad, LPHEA-Marrakech, Marrakech, Morocco; Faculté des Sciences, Université Mohamed Premier and LPTPM, Oujda, Morocco; Faculté des Sciences, Université Mohammed V-Agdal, Rabat, Morocco; DSM/IRFU (Institut de Recherches sur les Lois Fondamentales de l’Univers), CEA Saclay (Commissariat à l’Energie Atomique et aux Energies Alternatives), Gif-sur-Yvette, France; Santa Cruz Institute for Particle Physics, University of California Santa Cruz, Santa Cruz, CA USA; Department of Physics, University of Washington, Seattle, WA USA; Department of Physics and Astronomy, University of Sheffield, Sheffield, UK; Department of Physics, Shinshu University, Nagano, Japan; Fachbereich Physik, Universität Siegen, Siegen, Germany; Department of Physics, Simon Fraser University, Burnaby, BC Canada; SLAC National Accelerator Laboratory, Stanford, CA USA; Faculty of Mathematics, Physics and Informatics, Comenius University, Bratislava, Slovak Republic; Department of Subnuclear Physics, Institute of Experimental Physics of the Slovak Academy of Sciences, Kosice, Slovak Republic; Department of Physics, University of Cape Town, Cape Town, South Africa; Department of Physics, University of Johannesburg, Johannesburg, South Africa; School of Physics, University of the Witwatersrand, Johannesburg, South Africa; Department of Physics, Stockholm University, Stockholm, Sweden; The Oskar Klein Centre, Stockholm, Sweden; Physics Department, Royal Institute of Technology, Stockholm, Sweden; Departments of Physics and Astronomy and Chemistry, Stony Brook University, Stony Brook, NY USA; Department of Physics and Astronomy, University of Sussex, Brighton, UK; School of Physics, University of Sydney, Sydney, Australia; Institute of Physics, Academia Sinica, Taipei, Taiwan; Department of Physics, Technion: Israel Institute of Technology, Haifa, Israel; Raymond and Beverly Sackler School of Physics and Astronomy, Tel Aviv University, Tel Aviv, Israel; Department of Physics, Aristotle University of Thessaloniki, Thessaloníki, Greece; International Center for Elementary Particle Physics and Department of Physics, The University of Tokyo, Tokyo, Japan; Graduate School of Science and Technology, Tokyo Metropolitan University, Tokyo, Japan; Department of Physics, Tokyo Institute of Technology, Tokyo, Japan; Department of Physics, University of Toronto, Toronto, ON Canada; TRIUMF, Vancouver, BC, Canada; Department of Physics and Astronomy, York University, Toronto, ON Canada; Faculty of Pure and Applied Sciences, University of Tsukuba, Tsukuba, Japan; Department of Physics and Astronomy, Tufts University, Medford, MA USA; Centro de Investigaciones, Universidad Antonio Narino, Bogotá, Colombia; Department of Physics and Astronomy, University of California Irvine, Irvine, CA USA; INFN Gruppo Collegato di Udine, Sezione di Trieste, Udine, Italy; ICTP, Trieste, Italy; Dipartimento di Chimica, Fisica e Ambiente, Università di Udine, Udine, Italy; Department of Physics, University of Illinois, Urbana, IL USA; Department of Physics and Astronomy, University of Uppsala, Uppsala, Sweden; Instituto de Física Corpuscular (IFIC) and Departamento de Física Atómica, Molecular y Nuclear and Departamento de Ingeniería Electrónica and Instituto de Microelectrónica de Barcelona (IMB-CNM), University of Valencia and CSIC, Valencia, Spain; Department of Physics, University of British Columbia, Vancouver, BC Canada; Department of Physics and Astronomy, University of Victoria, Victoria, BC Canada; Department of Physics, University of Warwick, Coventry, UK; Waseda University, Tokyo, Japan; Department of Particle Physics, The Weizmann Institute of Science, Rehovot, Israel; Department of Physics, University of Wisconsin, Madison, WI USA; Fakultät für Physik und Astronomie, Julius-Maximilians-Universität, Würzburg, Germany; Fachbereich C Physik, Bergische Universität Wuppertal, Wuppertal, Germany; Department of Physics, Yale University, New Haven, CT USA; Yerevan Physics Institute, Yerevan, Armenia; Centre de Calcul de l’Institut National de Physique Nucléaire et de Physique des Particules (IN2P3), Villeurbanne, France; CERN, 1211 Geneva 23, Switzerland

## Abstract

Results of a search for new phenomena in final states with an energetic jet and large missing transverse momentum are reported. The search uses 20.3 fb${}^{-1}$ of $\sqrt{s}=8$ TeV data collected in 2012 with the ATLAS detector at the LHC. Events are required to have at least one jet with $p_{\mathrm {T}}> 120$ GeV and no leptons. Nine signal regions are considered with increasing missing transverse momentum requirements between $E_{\mathrm {T}}^{\mathrm {miss}}>150$ GeV and $E_{\mathrm {T}}^{\mathrm {miss}}> 700$ GeV. Good agreement is observed between the number of events in data and Standard Model expectations. The results are translated into exclusion limits on models with either large extra spatial dimensions, pair production of weakly interacting dark matter candidates, or production of very light gravitinos in a gauge-mediated supersymmetric model. In addition, limits on the production of an invisibly decaying Higgs-like boson leading to similar topologies in the final state are presented.

## Introduction

Events with an energetic jet and large missing transverse momentum in the final state constitute a clean and distinctive signature in searches for new physics beyond the Standard Model (SM) at colliders. Such signatures are referred to as monojet-like in this paper. In particular, monojet-like (as well as monophoton and mono-*W* / *Z*) final states have been studied [1–21] in the context of searches for supersymmetry (SUSY), large extra spatial dimensions (LED), and the search for weakly interacting massive particles (WIMPs) as candidates for dark matter (DM).

The Arkani-Hamed, Dimopoulos, and Dvali (ADD) model for LED [22] explains the large difference between the electroweak unification scale at $O(10^2$) GeV and the Planck scale $M_\mathrm{{Pl}} \sim O(10^{19}$) GeV by postulating the presence of *n* extra spatial dimensions of size *R*, and defining a fundamental Planck scale in $4+n$ dimensions, $M_D$, given by ${M_\mathrm{{Pl}}}^2 \sim {M_D}^{2+n}R^n$. An appropriate choice of *R* for a given *n* yields a value of $M_D$ at the electroweak scale. The extra spatial dimensions are compactified, resulting in a Kaluza–Klein tower of massive graviton modes. If produced in high-energy collisions in association with an energetic jet, these graviton modes escape detection leading to a monojet-like signature in the final state.

A non-baryonic DM component in the universe is commonly used to explain a range of astrophysical measurements (see, for example, Ref. [23] for a review). Since none of the known SM particles are adequate DM candidates, the existence of a new particle is often hypothesized. Weakly interacting massive particles are one such class of particle candidates that can be searched for at the LHC [24]. They are expected to couple to SM particles through a generic weak interaction, which could be the weak interaction of the SM or a new type of interaction. Such a new particle would result in the correct relic density values for non-relativistic matter in the early universe [25], as measured by the PLANCK [26] and WMAP [27] satellites, if its mass is between a few GeV and a TeV and if it has electroweak-scale interaction cross sections. Many new particle physics models such as SUSY [28–36] also predict WIMPs.

Because WIMPs interact so weakly that they do not deposit energy in the calorimeter, their production leads to signatures with missing transverse momentum. Here, WIMPs are assumed to be produced in pairs, and the events are identified via the presence of an energetic jet from initial-state radiation (ISR) [37–40] yielding large missing transverse momentum.

**Fig. 1:**
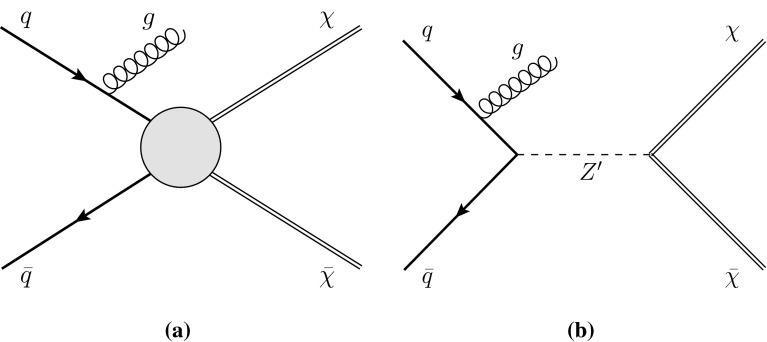
Feynman diagrams for the production of weakly interacting massive particle pairs $\chi \bar{\chi }$ associated with a jet from initial-state radiation of a gluon, *g*. **a** A contact interaction described with effective operators. **b** A simplified model with a $Z^\prime$ boson

**Table 1:** Effective interactions coupling WIMPs to Standard Model quarks or gluons, following the formalism in Ref. [41], where $M_\star$ is the suppression scale of the interaction. Operators starting with a D describe Dirac fermion WIMPs, the ones starting with a C are for scalar WIMPs and $G^a_{\mu \nu }$ is the colour field-strength tensor

Name	Initial state	Type	Operator
C1	*qq*	Scalar	$\frac{m_q}{M^2_\star }{\chi ^\dagger }\chi \bar{q}q$
C5	*gg*	Scalar	$\frac{1}{4M^2_\star }{\chi ^\dagger }\chi \alpha _\mathrm{s}(G^a_{\mu \nu })^2$
D1	*qq*	Scalar	$\frac{m_q}{M^3_\star }\bar{\chi }\chi \bar{q}q$
D5	*qq*	Vector	$\frac{1}{M^2_\star }\bar{\chi }\gamma ^{\mu }\chi \bar{q}\gamma _{\mu } q$
D8	*qq*	Axial-vector	$\frac{1}{M^2_\star }\bar{\chi }\gamma ^{\mu }\gamma ^5\chi \bar{q}\gamma _{\mu }\gamma ^5 q$
D9	*qq*	Tensor	$\frac{1}{M_\star ^2}\bar{\chi }\sigma ^{\mu \nu }\chi \bar{q}\sigma _{\mu \nu }q$
D11	*gg*	Scalar	$\frac{1}{4M_\star ^3}\bar{\chi }\chi \alpha _\mathrm{s}(G^a_{\mu \nu })^2$

The interaction of WIMPs with SM particles is described as a contact interaction using an effective field theory (EFT) approach, mediated by a single new heavy particle or particles with mass too large to be produced directly at the LHC (see Fig. 1a). It is assumed here that the DM particle is either a Dirac fermion or a scalar $\chi$; the only difference for Majorana fermions is that certain interactions are not allowed and that the cross sections for the allowed interactions are larger by a factor of four. Seven interactions are considered (see Table 1), namely those described by the operators C1, C5, D1, D5, D8, D9, D11, following the naming scheme in Ref. [41]. These operators describe different bilinear quark couplings to WIMPs, $q\bar{q}\rightarrow \chi \bar{\chi }$, except for C5 and D11, which describe the coupling to gluons, $gg\rightarrow \chi \bar{\chi }$. The operators for Dirac fermions and scalars in Ref. [41] fall into six categories with characteristic missing transverse momentum spectral shapes. The representative set of operators for these six categories are C1, C5, D1, D5, D9, and D11, while D8 falls into the same category as D5 but is listed explicitly in Table 1 because it is often used to convert LHC results into limits on DM pair production. In the operator definitions in Table 1, $M_*$ is the suppression scale of the interaction, after integrating out the heavy mediator particles. The use of a contact interaction to produce WIMP pairs via heavy mediators is considered conservative because it rarely overestimates cross sections when applied to a specific scenario for physics beyond the SM. Cases where this approach is indeed optimistic are studied in Refs. [40, 42–46]. Despite the caveats related to the validity of the EFT approach (see Appendix A), this formalism is used here, as it provides a framework for comparing LHC results to existing direct or indirect DM searches. Within this framework, interactions of SM and DM particles are described by only two parameters, the suppression scale ${M}_\star$ and the DM particle mass $m_\chi$. Besides the EFT operators, the pair production of WIMPs is also investigated within a so-called simplified model, where a pair of WIMPs couples to a pair of quarks explicitly via a new mediator particle, a new vector boson $Z^\prime$ (see Fig. 1b).

In gauge-mediated SUSY-breaking (GMSB) scenarios [47–52], the gravitino $\tilde{G}$ (spin-3/2 superpartner of the graviton) is often the lightest supersymmetric particle and a potential candidate for DM. Its mass is related to the SUSY-breaking scale $\sqrt{F}$ and $M_\mathrm{{Pl}}$ via $m_{\tilde{G}}\propto F/M_\mathrm{{Pl}}$ [53]. At hadron colliders, in low-scale SUSY-breaking scenarios with very light gravitinos, the cross section for associated production of gravitino–squark ($pp \rightarrow \tilde{G}\tilde{q}+ X$) and gravitino–gluino ($pp \rightarrow \tilde{G}\tilde{g}+ X$) processes are relatively large [54], since the cross section depends on $m_{\tilde{G}}$ as $\sigma \sim 1/m_{\tilde{G}}^2$. The decay of the gluino or squark into a gravitino and a gluon ($\tilde{g}\rightarrow \tilde{G}g$) or a gravitino and a quark ($\tilde{q}\rightarrow \tilde{G}q$), respectively, dominates [54]. The final state is characterized by the presence of a pair of gravitinos that escape detection and an energetic jet, leading to a monojet-like topology. Previous studies at colliders [16, 55] considered the production of gravitinos in association with a photon or a jet and assumed extremely heavy squarks and gluinos. Within this approximation, a lower limit for the gravitino mass of $m_{\tilde{G}}> 1.37 \times 10^{-5}$ eV was established.

The study of the properties of the Higgs boson discovered by the ATLAS and CMS experiments [56, 57] does not exclude a sizeable branching ratio for its decay to invisible particles. It also opens up the question of whether a Higgs-like scalar field plays an important role in describing the interaction between dark and ordinary matter in the universe. In particular, a sizeable branching ratio to invisible particles could be interpreted in terms of the production of DM. Results from LEP [58] excluded an invisibly decaying Higgs boson, produced in association with a *Z* boson, for a boson mass ($m_{H}$) below 114.4 GeV. The strongest direct bounds from the LHC experiments on the branching ratio for the Higgs invisible decay mode [59, 60] set upper limits of 58 $\%$–65 $\%$ at 95 $\%$ confidence level (CL), based on the final state in which the Higgs boson is produced either in association with a *Z* boson or via vector-boson fusion processes. In this analysis, the monojet-like final state is used to search for the production of an invisibly decaying boson with SM Higgs-like properties and a mass in the range between 115 GeV and 300 GeV.

The paper is organized as follows. The ATLAS detector is described in the next section. Section 3 provides details of the simulations used in the analysis for background and signal processes. Section 4 discusses the reconstruction of jets, leptons and missing transverse momentum, while Sect. 5 describes the event selection. The estimation of background contributions and the study of systematic uncertainties are discussed in Sects. 6 and 7. The results are presented in Sect. 8, and are interpreted in terms of the search for ADD LED, WIMP pair production, the production of very light gravitinos in GMSB scenarios, and the production of an invisibly decaying Higgs-like boson. Finally, Sect. 9 is devoted to the conclusions.

## Experimental setup

The ATLAS detector [61] covers almost the whole solid angle^1^ around the collision point with layers of tracking detectors, calorimeters and muon chambers. The ATLAS inner detector (ID) has full coverage in $\phi$ and covers the pseudorapidity range $|\eta |<2.5$. It consists of a silicon pixel detector, a silicon microstrip detector, and a straw tube tracker which also measures transition radiation for particle identification, all immersed in a 2 T axial magnetic field produced by a solenoid.

High-granularity liquid-argon (LAr) electromagnetic sampling calorimeters, with excellent energy and position resolution, cover the pseudorapidity range $|\eta |<$ 3.2. The hadronic calorimetry in the range $|\eta |<$ 1.7 is provided by a scintillator-tile calorimeter, consisting of a large barrel and two smaller extended barrel cylinders, one on either side of the central barrel. In the endcaps ($|\eta |>$ 1.5), LAr hadronic calorimeters match the outer $|\eta |$ limits of the endcap electromagnetic calorimeters. The LAr forward calorimeters provide both the electromagnetic and hadronic energy measurements, and extend the coverage to $|\eta | < 4.9$.

The muon spectrometer measures the deflection of muons in the magnetic field provided by large superconducting air-core toroid magnets in the pseudorapidity range $|\eta |<2.7$, instrumented with separate trigger and high-precision tracking chambers. Over most of the $\eta$ range, a measurement of the track coordinates in the principal bending direction of the magnetic field is provided by monitored drift tubes. At large pseudorapidities, cathode strip chambers with higher granularity are used in the innermost plane over $2.0 < |\eta | < 2.7$. The muon trigger system covers the pseudorapidity range $|\eta | < 2.4$.

The data are collected using an online three-level trigger system [62] that selects events of interest and reduces the event rate from several MHz to about 400 Hz for recording and offline processing.

## Monte Carlo simulation

Simulated event samples are used to compute detector acceptance and reconstruction efficiencies, determine signal and background contributions, and estimate systematic uncertainties in the final results.

### Background simulation

The expected background to the monojet-like signature is dominated by $Z(\rightarrow \nu \bar{\nu })+$jets and $W+$jets production (with $W(\rightarrow \tau \nu )+$jets being the dominant among the $W+$jets backgrounds), and includes small contributions from $Z/\gamma ^*(\rightarrow \ell ^+ \ell ^-)+$jets ($\ell = e, \mu , \tau$), multijet, $t\bar{t}$, single-top, and diboson ($WW,WZ,ZZ,W\gamma ,Z\gamma$) processes.

Samples of simulated $W+$jets and $Z+$jets production events are generated using SHERPA-1.4.1 [63] Monte Carlo (MC) generator, including leading-order (LO) matrix elements for up to five partons in the final state and assuming massive *b*/*c*-quarks, with CT10 [64] parton distribution functions (PDF) of the proton. The MC expectations are initially normalized to next-to-next-to-leading-order (NNLO) perturbative QCD (pQCD) predictions according to DYNNLO [65, 66] using MSTW2008 90 $\%$ CL NNLO PDF sets [67]. The production of top-quark pairs ($t\bar{t}$) is simulated using the MC@NLO-4.06 [68, 69] MC generator with parton showers and underlying-event modelling as implemented in HERWIG-6.5.20 [70, 71] plus JIMMY [72]. Single-top production samples are generated with MC@NLO [73] for the *s*- and *Wt*-channel [74], while AcerMC-v3.8 [75] is used for single-top production in the *t*-channel. A top-quark mass of 172.5 $\,\hbox {GeV}$ is used consistently. The AUET2C and AUET2B [76] set of optimised parameters for the underlying event description are used for $t\bar{t}$ and single-top processes, which use CT10 and CTEQ6L1 [77] PDF, respectively. Approximate NNLO$+$NNLL (next-to-next-to-leading-logarithm) pQCD cross sections, as determined in TOP$++$2.0 [78], are used in the normalization of the $t\bar{t}$  [79] and *Wt* [80] samples. Multijet and $\gamma +$jet samples are generated using the PYTHIA-8.165 program [81] with CT10 PDF. Finally, diboson samples (*WW*, *WZ*, *ZZ*, $W\gamma$ and $Z\gamma$ production) are generated using SHERPA with CT10 PDF and are normalized to NLO pQCD predictions [82].

### Signal simulation

Simulated samples for the ADD LED model with different number of extra dimensions in the range $n=2$–6 and $M_D$ in the range 2–5 TeV are generated using PYTHIA-8.165 with CT10 PDF. Renormalization and factorization scales are set to $\sqrt{1/2 \times m_{G}^2 + p_\mathrm{T}^2}$, where $m_G$ is the graviton mass and $p_\mathrm{T}$ denotes the transverse momentum of the recoiling parton.

The effective field theory of WIMP pair production is implemented in MADGRAPH5-v1.5.2 [83], taken from Ref. [41]. The WIMP pair production plus one or two additional partons from ISR is simulated in two ways. For all operators, samples are generated requiring at least one parton with a minimum $p_{\mathrm {T}}$ of $80~\,\hbox {GeV}$. Studies simulating up to three additional partons along with the WIMP pair showed no difference in kinematic distributions when compared to the samples with up to two additional partons.

Only initial states of gluons and the four lightest quarks are considered, assuming equal coupling strengths for all quark flavours to the WIMPs. The mass of the charm quark is most relevant for the cross sections of the operator D1 (see Table 1) and it is set to $1.42~\,\hbox {GeV}$. The generated events are interfaced to PYTHIA-6.426 [84] for parton showering and hadronization. The MLM prescription [85] is used for matching the matrix-element calculations of MADGRAPH5 to the parton shower evolution of PYTHIA-6. The samples are subsequently reweighted to the MSTW2008LO [67] PDF set using LHAPDF [86]. The MADGRAPH5 default choice for the renormalization and factorization scales is used. The scales are set to the geometric average of ${m^2 + p_{\mathrm {T}}^2}$ for the two WIMPs, where *m* is the mass of the particles. Events with WIMP masses between 10 GeV and 1300 GeV are simulated for six different effective operators (C1, C5, D1, D5, D9, D11). The WIMPs are taken to be either Dirac fermions (*D* operators) or scalars (*C* operators), and the pair-production cross section is calculated at LO. To study the transition between the effective field theory and a physical renormalizable model for Dirac fermion WIMPs coupling to Standard Model particles via a new mediator particle $Z^\prime$, a simplified model is generated in MADGRAPH5. For each WIMP mass point, mediator particle masses ${M}_\mathrm {med}$ between 50 GeV and 30 TeV are considered, each for two values of the mediator particle width ($\Gamma ={M}_\mathrm {med}/3$ and ${M}_\mathrm {med}/8\pi$).

Simulated samples for gravitino production in association with a gluino or a squark in the final state, $pp \rightarrow \tilde{G}\tilde{g} + X$ and $pp \rightarrow \tilde{G}\tilde{q} + X$, are generated using LO matrix elements in MADGRAPH4.4 [87] interfaced with PYTHIA-6.426 and using CTEQ6L1 PDF. The narrow-width approximation for the gluino and squark decays $\tilde{g} \rightarrow g \tilde{G}$ and $\tilde{q} \rightarrow q \tilde{G}$ is assumed. The renormalization and factorization scales are set to the average of the mass of the final-state particles involved in the hard interaction $(m_{\tilde{G}}+ m_{\tilde{q}/\tilde{g}})/2 \simeq m_{\tilde{q}/\tilde{g}}/2$. Values for $m_{\tilde{G}}$ in the range between $10^{-3}$ eV and $10^{-5}$ eV are considered for squark and gluino masses in the range 50 GeV to 2.6 TeV.

Finally, simulated samples for the production of a Higgs boson are generated including the $gg \rightarrow H$, $VV \rightarrow H$ ($V = W,Z$), and *VH* production channels. Masses for the boson in the range between 115 GeV and 300 GeV are considered. This Higgs boson is assumed to be produced as predicted in the Standard Model but unlike the SM Higgs it may decay into invisible particles at a significant rate. The signal is modelled using POWHEG-r2262 [88–90], which calculates separately the $gg \rightarrow H$ and $VV \rightarrow H$ production mechanisms with NLO pQCD matrix elements. The description of the Higgs boson $p_{\mathrm {T}}$ spectrum in the $gg \rightarrow H$ process follows the calculation in Ref. [91], which includes NLO $+$ NNLL corrections. The effects of finite quark masses are also taken into account [92]. For $gg \rightarrow H$ and $VV \rightarrow H$ processes, POWHEG is interfaced to PYTHIA-8.165 for showering and hadronization. For *ZH* and *WH* processes, POWHEG interfaced to HERWIG$++$ [93] is used and the *Z* / *W* bosons are forced to decay to a pair of quarks. The invisible decay of the Higgs-like boson is simulated by forcing the boson to decay to two *Z* bosons, which are then forced to decay to neutrinos. Signal samples are generated with renormalization and factorization scales set to $\sqrt{(m_{H})^2 + (p_\mathrm{T}^{H})^2}$. The Higgs boson production cross sections, as well as their uncertainties, are taken from Refs. [94, 95]. For the $gg \rightarrow H$ process, cross-section calculations at NNLO$+$NNLL accuracy  [96–99] in pQCD are used and NLO electroweak corrections [100, 101] are included. The cross sections for $VV \rightarrow H$ processes are calculated with full NLO pQCD and electroweak corrections [102–104]. The cross sections for the associated production (*WH* and *ZH*) are calculated at NNLO [105] in pQCD, and include NLO electroweak corrections [106].

Differing pileup (multiple proton–proton interactions in the same or neighbouring bunch-crossings) conditions as a function of the instantaneous luminosity are taken into account by overlaying simulated minimum-bias events generated with PYTHIA-8 onto the hard-scattering process. The MC-generated samples are processed either with a full ATLAS detector simulation [107] based on the GEANT4 program [108] or a fast simulation of the response of the electromagnetic and hadronic calorimeters [109] and of the trigger system. The results based on fast simulation are validated against fully simulated samples and the difference is found to be negligible. The simulated events are reconstructed and analysed with the same analysis chain as for the data, using the same trigger and event selection criteria.

## Reconstruction of physics objects

Jets are defined using the $\hbox {anti}-{k_{t}}$ jet algorithm [110] with the radius parameter $R = 0.4$. Energy depositions reconstructed as clusters in the calorimeter are the inputs to the jet algorithm. The measured jet $p_{\mathrm {T}}$ is corrected for detector effects, including the non-compensating character of the calorimeter, by weighting energy deposits arising from electromagnetic and hadronic showers differently. In addition, jets are corrected for contributions from pileup, as described in Ref. [111]. Jets with corrected $p_\mathrm{T} > 30$ GeV and $|\eta | <4.5$ are considered in the analysis. Jets with $|\eta | < 2.5$ containing a *b*-hadron are identified using a neural-net-based algorithm [112] with an efficiency of 80$\%$ and a rejection factor of 30 (3) against jets originating from fragmentation of light quarks or gluons (jets containing a *c*-hadron), as determined using simulated $t\bar{t}$ events.

The presence of leptons (muons or electrons) in the final state is used in the analysis to define control samples and to reject background contributions in the signal regions (see Sects. 5,  6). Muon candidates are formed by combining information from the muon spectrometer and inner tracking detectors as described in Ref. [113] and are required to have $p_\mathrm{T} > 7$ GeV and $|\eta | < 2.5$. In addition, muons are required to be isolated: the sum of the transverse momenta of the tracks not associated with the muon in a cone of size $\Delta R=\sqrt{(\Delta \eta )^2 + (\Delta \phi )^2} = 0.2$ around the muon direction is required to be less than 1.8 GeV. The muon $p_{\mathrm {T}}$ requirement is increased to $p_{\mathrm {T}}> 20$ GeV to define the $W(\rightarrow \mu \nu )+$jets and $Z/\gamma ^*(\rightarrow \mu ^+\mu ^-)+$jets control regions.

Electron candidates are initially required to have $p_\mathrm{T} > 7$ GeV and $|\eta | <2.47$, and to pass the medium electron shower shape and track selection criteria described in Ref. [114], which are reoptimized for 2012 data. Overlaps between identified electrons and jets in the final state are resolved. Jets are discarded if their separation $\Delta R$ from an identified electron is less than 0.2. The electron $p_{\mathrm {T}}$ requirement is increased to $p_{\mathrm {T}}> 20$ GeV and the transition region between calorimeter sections $1.37 < |\eta | < 1.52$ is excluded to reconstruct *Z* and *W* boson candidates in the $Z/\gamma ^*(\rightarrow e^+e^-)+$jets and $W(\rightarrow e \nu )+$jets control regions, respectively. The electron requirements are further tightened for the $W(\rightarrow e \nu )+$jets control sample to constrain the irreducible $Z(\rightarrow \nu \bar{\nu })+$jets background contribution (see below). In this case, electrons are selected to pass tight [114] electron shower shape and track selection criteria, their $p_{\mathrm {T}}$ threshold is raised to 25 GeV, and they are required to be isolated: the sum of the transverse momenta of the tracks not associated with the electron in a cone of radius $\Delta R = 0.3$ around the electron direction is required to be less than 5 $\%$ of the electron $p_{\mathrm {T}}$. An identical isolation criterion, based on the calorimeter energy deposits not associated with the electron, is also applied.

The missing transverse momentum (${{\mathbf {p}}_\mathrm{T}^\mathrm{\ miss}}$), the magnitude of which is called $E_{\mathrm {T}}^{\mathrm {miss}}$, is reconstructed using all energy deposits in the calorimeter up to pseudorapidity $|\eta | = 4.9$. Clusters associated with either electrons or photons with $p_{\mathrm {T}}>10$ GeV and those associated with jets with $p_{\mathrm {T}}>20$ GeV make use of the corresponding calibrations for these objects. Softer jets and clusters not associated with these objects are calibrated using both calorimeter and tracking information [115].

## Event selection

The data sample considered in this paper corresponds to a total integrated luminosity of $20.3 \ \mathrm fb^{-1}$. The uncertainty in the integrated luminosity is $2.8~\%$, as estimated following the same methodology as detailed in Ref. [116]. The data were selected online using a trigger logic that selects events with $E_{\mathrm {T}}^{\mathrm {miss}}$ above 80 GeV, as computed at the final stage of the three-level trigger system [62]. With respect to the final analysis requirements, the trigger selection is fully efficient for $E_{\mathrm {T}}^{\mathrm {miss}}> 150$ GeV, as determined using a data sample with muons in the final state. Table 2 summarizes the different event selection criteria applied in the signal regions. The following preselection criteria are applied.

- Events are required to have a reconstructed primary vertex for the interaction consistent with the beamspot envelope and to have at least two associated tracks with $p_{\mathrm {T}}> 0.4$ GeV; when more than one such vertex is found, the vertex with the largest summed $p_{\mathrm {T}}^2$ of the associated tracks is chosen.
- Events are required to have $E_{\mathrm {T}}^{\mathrm {miss}}> 150$ GeV and at least one jet with $p_\mathrm{T}> 30$ GeV and $|\eta | < 4.5$ in the final state.
- The analysis selects events with a leading jet with $p_{\mathrm {T}}> 120$ GeV and $|\eta | < 2.0$. Monojet-like topologies in the final state are selected by requiring the leading-jet $p_{\mathrm {T}}$ and the $E_{\mathrm {T}}^{\mathrm {miss}}$ to satisfy $p_{\mathrm {T}}/E_{\mathrm {T}}^{\mathrm {miss}}> 0.5$. An additional requirement on the azimuthal separation $\Delta \phi (\text {jet},{{\mathbf {p}}_\mathrm{T}^\mathrm{\ miss}}) > 1.0$ between the direction of the missing transverse momentum and that of each of the selected jets is imposed. This requirement reduces the multijet background contribution where the large $E_{\mathrm {T}}^{\mathrm {miss}}$ originates mainly from jet energy mismeasurement.
- Events are rejected if they contain any jet with $p_\mathrm{T}> 20$ GeV and $|\eta | < 4.5$ that presents an electromagnetic fraction in the calorimeter, calorimeter sampling fraction, or charged fraction^2^ (for jets with $|\eta |<2.5$) inconsistent with the requirement that they originate from a proton–proton collision [117]. In the case of the leading (highest $p_{\mathrm {T}}$) jet in the event, the requirements are tightened to reject remaining contributions from beam-related backgrounds and cosmic rays. Events are also rejected if any of the jets is reconstructed close to known partially instrumented regions of the calorimeter. Additional requirements based on the timing and the pulse shape of the cells in the calorimeter are applied to suppress coherent noise and electronic noise bursts in the calorimeter producing anomalous energy deposits [118]; these requirements have a negligible effect on the signal efficiency.
- Events with muons or electrons with $p_{\mathrm {T}}> 7$ GeV are vetoed. In addition, events with isolated tracks with $p_{\mathrm {T}}> 10$ GeV and $|\eta | < 2.5$ are vetoed to reduce background from non-identified leptons (*e*, $\mu$ or $\tau$) in the final state. The track isolation is defined such that there must be no additional track with $p_{\mathrm {T}}> 3$ GeV within a cone of radius 0.4 around it.

Different signal regions (SR1–SR9) are considered with increasing $E_{\mathrm {T}}^{\mathrm {miss}}$ thresholds from 150 GeV to 700 GeV.

**Table 2:** Event selection criteria applied for the selection of monojet-like signal regions, SR1–SR9

Selection criteria									
Preselection									
Primary vertex									
$E_{\mathrm {T}}^{\mathrm {miss}}> 150$ GeV									
Jet quality requirements									
At least one jet with $p_{\mathrm {T}}>30$ GeV and $|\eta |< 4.5$									
Lepton and isolated track vetoes									
Monojet-like selection									
The leading jet with $p_{\mathrm {T}}> 120$ GeV and $|\eta |<2.0$									
Leading jet $p_{\mathrm {T}}/E_{\mathrm {T}}^{\mathrm {miss}}> 0.5$									
$$\Delta \phi (\text {jet},{\mathbf {p}}_\mathrm{T}^\mathrm{\ miss}) > 1.0$$									
Signal region	SR1	SR2	SR3	SR4	SR5	SR6	SR7	SR8	SR9
Minimum $E_{\mathrm {T}}^{\mathrm {miss}}$ (GeV)	150	200	250	300	350	400	500	600	700

## Background estimation

The $W+$jets and $Z(\rightarrow \nu \bar{\nu })+$jets backgrounds are estimated using MC event samples normalized using data in selected control regions. In particular, the dominant $Z(\rightarrow \nu \bar{\nu })+$jets background contribution is constrained using a combination of estimates from $W+$jets and $Z+$jets control regions. The remaining SM backgrounds from $Z/\gamma ^*(\rightarrow \ell ^+ \ell ^-)+$jets, $t\bar{t}$, single top, and dibosons are determined using simulated samples, while the multijet background contribution is extracted from data. In the case of the $t\bar{t}$ background process, which contributes to both the signal and $W+$jets control regions, dedicated control samples are defined to validate the MC normalization and to estimate systematic uncertainties. Finally, the potential contributions from beam-related background and cosmic rays are estimated in data using jet timing information. The methodology and the samples used for estimating the background are summarised in Table 3. The details are given in the following sections.

**Table 3:** Summary of the methods and control samples used to constrain the different background contributions in the signal regions

Background process	Method	Control sample
$Z(\rightarrow \nu \bar{\nu })+$jets	MC and control samples in data	$Z/\gamma ^*(\rightarrow \ell ^+ \ell ^-)$, $W(\rightarrow \ell \nu )$ ($\ell = e,\mu$)
$W(\rightarrow e \nu )+$jets	MC and control samples in data	$W(\rightarrow e \nu )$ (loose)
$W(\rightarrow \tau \nu )+$jets	MC and control samples in data	$W(\rightarrow e \nu )$ (loose)
$W(\rightarrow \mu \nu )+$jets	MC and control samples in data	$W(\rightarrow \mu \nu )$
$Z/\gamma ^*(\rightarrow \ell ^+ \ell ^-)+$jets ($\ell = e,\mu ,\tau$)	MC-only	
$t\bar{t}$, single top	MC-only	
Diboson	MC-only	
Multijets	Data-driven	
Non-collision	Data-driven	

### $W/Z+$jets background

Control samples in data, with identified electrons or muons in the final state and with identical requirements on the jet $p_\mathrm{T}$ and $E_{\mathrm {T}}^{\mathrm {miss}}$, are used to determine the $W(\rightarrow \ell \nu )+$jets ($\ell = e,\mu ,\tau$) and $Z(\rightarrow \nu \bar{\nu })+$jets electroweak background contributions. This reduces significantly the relatively large theoretical and experimental systematic uncertainties, of the order of $20~\%$–$40~\%$, associated with purely MC-based expectations. The $E_{\mathrm {T}}^{\mathrm {miss}}$-based online trigger used does not include muon information in the $E_{\mathrm {T}}^{\mathrm {miss}}$ calculation. This allows the collection of $W(\rightarrow \mu \nu )+$jets and $Z/\gamma ^*(\rightarrow \mu ^+\mu ^-)+$jets control samples with the same trigger as for the signal regions. This is not the case for the $W(\rightarrow e \nu )+$jets and $Z/\gamma ^*(\rightarrow e^+e^-)+$jets control samples used to help with constraining the $Z(\rightarrow \nu \bar{\nu })+$jets background (see below).

A $W(\rightarrow \mu \nu )+$jets control sample is defined using events with a muon with $p_{\mathrm {T}}>20$ GeV and *W* transverse mass in the range 40 GeV $< m_\mathrm{T} < 100$ GeV. The transverse mass $m_\mathrm{T}$ is defined by the lepton ($\ell$) and neutrino ($\nu$) $p_{\mathrm {T}}$ and direction as $m_T=\sqrt{2p_{\mathrm {T}}^{\ell }p_{\mathrm {T}}^{\nu }(1-\cos (\phi ^{\ell }-\phi ^{\nu }))}$, where the (*x*, *y*) components of the neutrino momentum are taken to be the same as the corresponding ${\mathbf {p}}_\mathrm{T}^\mathrm{\ miss}$ components. Similarly, a $Z/\gamma ^*(\rightarrow \mu ^+\mu ^-)+$jets control sample is selected, requiring the presence of two muons with $p_{\mathrm {T}}>20$ GeV and invariant mass in the range 66 GeV $< m_{\mu \mu } < 116$ GeV. In the $W(\rightarrow \mu \nu )+$jets and $Z/\gamma ^*(\rightarrow \mu ^+\mu ^-)+$jets control regions, the $E_{\mathrm {T}}^{\mathrm {miss}}$ is not corrected for the presence of the muons in the final state, which are considered invisible, motivated by the fact that these control regions are used to estimate the irreducible $Z(\rightarrow \nu \bar{\nu })+$jets background in the signal regions.

**Fig. 2:**
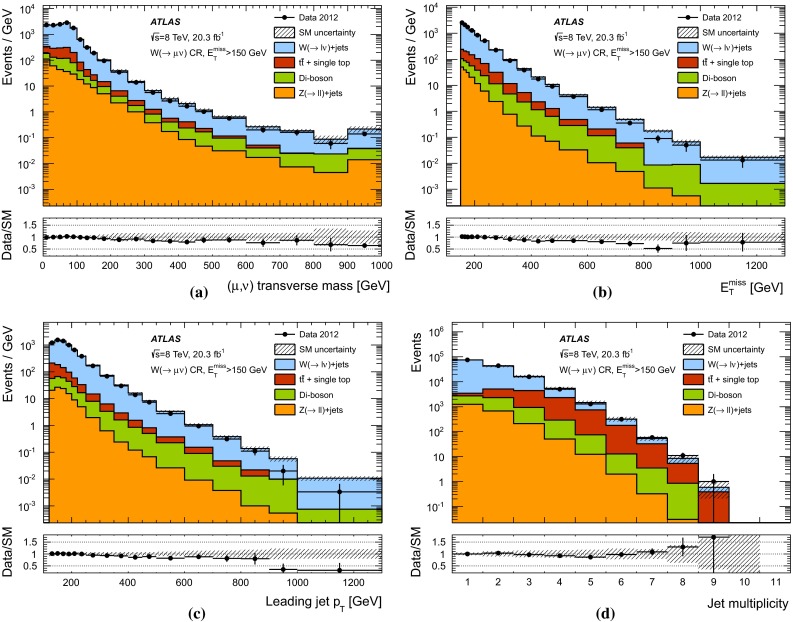
Distributions of the measured **a** transverse mass of the identified muon and the missing transverse momentum, **b** $E_{\mathrm {T}}^{\mathrm {miss}}$, **c** leading jet $p_{\mathrm {T}}$ and **d** jet multiplicity distributions in the $W(\rightarrow \mu \nu )+$jets control region for the inclusive SR1 selection, compared to the background expectations. The latter include the global normalization factors extracted from the data. Where appropriate, the last bin of the distribution includes overflows. The lower panels represent the ratio of data to MC expectations. The error bands in the ratios include the statistical and experimental uncertainties in the background expectations

**Fig. 3:**
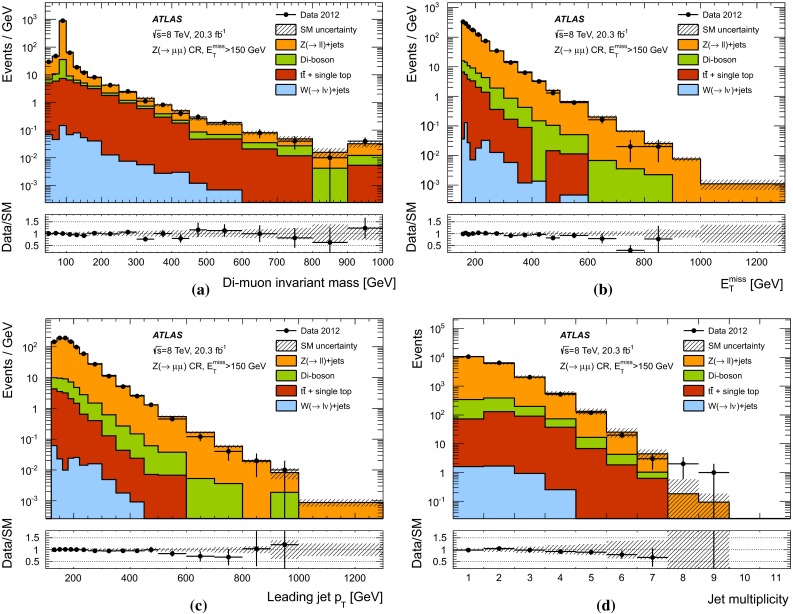
Distributions of the measured **a** dilepton invariant mass, **b** $E_{\mathrm {T}}^{\mathrm {miss}}$, **c** leading jet $p_{\mathrm {T}}$ and **d** jet multiplicity distributions in the $Z/\gamma ^*(\rightarrow \mu ^+\mu ^-)+$jets control region for the inclusive SR1 selection, compared to the background expectations. The latter include the global normalization factors extracted from the data. Where appropriate, the last bin of the distribution includes overflows. The *lower panels* represent the ratio of data to MC expectations. The *error bands* in the ratios include the statistical and experimental uncertainties in the background expectations

The $W(\rightarrow e \nu )+$jets and $Z/\gamma ^*(\rightarrow e^+e^-)+$jets control samples used in constraining the $Z(\rightarrow \nu \bar{\nu })+$jets background in the signal regions are collected using online triggers that select events with an electron in the final state. The $E_{\mathrm {T}}^{\mathrm {miss}}$ is corrected by removing the contributions from the electron energy clusters in the calorimeters. In the $Z/\gamma ^*(\rightarrow e^+e^-)+$jets control sample, events are selected with exactly two electrons with $p_{\mathrm {T}}> 20$ GeV and dilepton invariant mass in the range 66 GeV $< m_{e e} < 116$ GeV. In the $W(\rightarrow e \nu )+$jets control sample a tight selection is applied: events are selected to have only a single electron with $p_{\mathrm {T}}> 25$ GeV, transverse mass in the range 40 GeV $< m_\mathrm{T} < 100$ GeV, and uncorrected $E_{\mathrm {T}}^{\mathrm {miss}}>25$ GeV. The latter requirements suppress background contamination from multijet processes where jets are misidentified as electrons.

A separate $W(\rightarrow e \nu )+$jets control sample, collected with the $E_{\mathrm {T}}^{\mathrm {miss}}$-based trigger and looser requirements that increase the number of events, is defined to constrain the $W(\rightarrow e \nu )+$jets and $W(\rightarrow \tau \nu )+$jets background contributions. In this case, the electron $p_{\mathrm {T}}$ requirement is reduced to $p_{\mathrm {T}}> 20$ GeV and no further cuts on electron isolation and $m_\mathrm{T}$ are applied. In addition, the $E_{\mathrm {T}}^{\mathrm {miss}}$ calculation in this case is not corrected for the presence of the electron or $\tau$ leptons in the final state, as they contribute to the calorimeter-based $E_{\mathrm {T}}^{\mathrm {miss}}$ calculation in the signal regions.

**Fig. 4:**
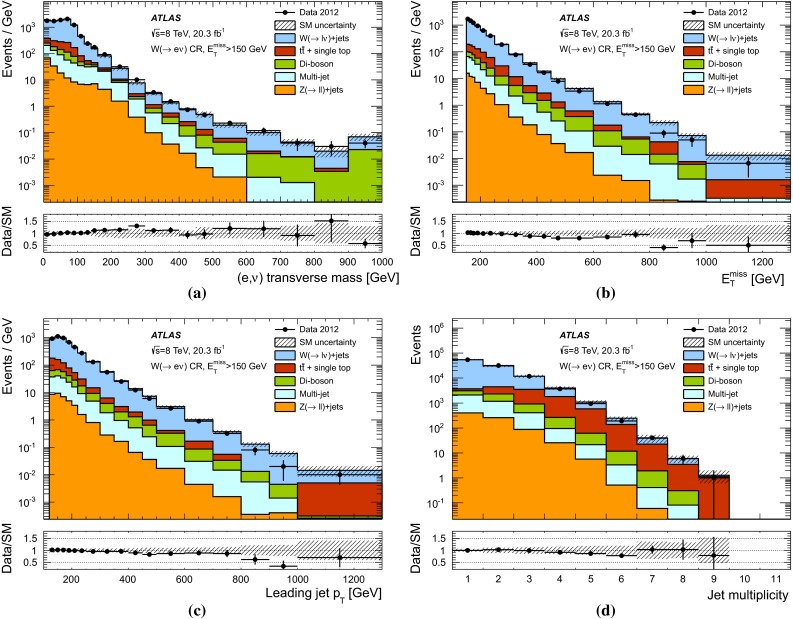
Distributions of the measured **a** transverse mass of the identified electron and the missing transverse momentum, **b** $E_{\mathrm {T}}^{\mathrm {miss}}$, **c** leading jet $p_{\mathrm {T}}$ and **d** jet multiplicity distributions in the $W(\rightarrow e \nu )+$jets control region for the inclusive SR1 selection, compared to the background expectations. The latter include the global normalization factors extracted from the data. Where appropriate, the last bin of the distribution includes overflows. The *lower panels* represent the ratio of data to MC expectations. The *error bands* in the ratios include the statistical and experimental uncertainties in the background expectations

**Fig. 5:**
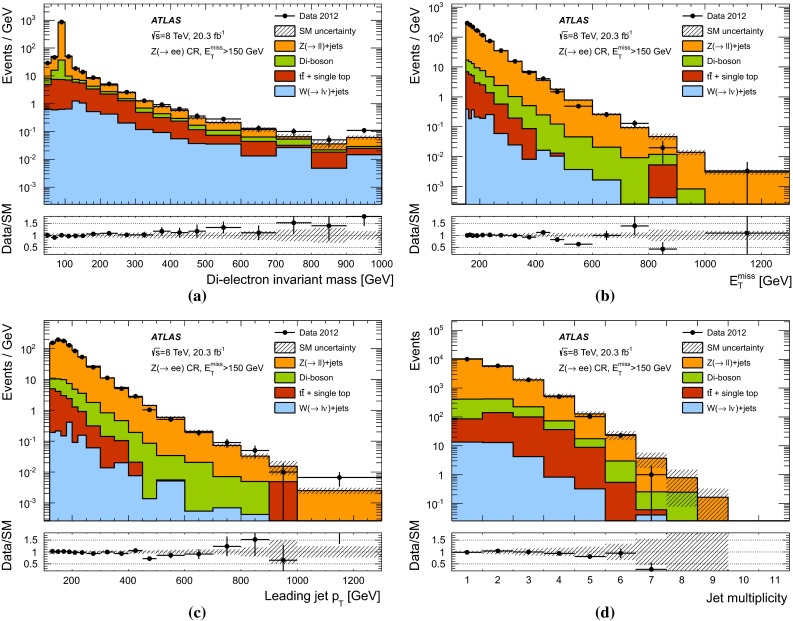
Distributions of the measured **a** dilepton invariant mass, **b** $E_{\mathrm {T}}^{\mathrm {miss}}$, **c** leading jet $p_{\mathrm {T}}$ and **d** jet multiplicity distributions in the $Z/\gamma ^*(\rightarrow e^+e^-)+$jets control region for the inclusive SR1 selection, compared to the background expectations. The latter include the global normalization factors extracted from the data. Where appropriate, the last bin of the distribution includes overflows. The *lower panels* represent the ratio of data to MC expectations. The *error bands* in the ratios include the statistical and experimental uncertainties in the background expectations

Figures 2, 3, 4 and 5 show some distributions in the different $W+$jets and $Z+$jets control regions in data compared to MC expectations for the SR1 monojet-like kinematic selection. In this case, the MC expectations are globally normalized to the data in the control regions, using normalization factors as explained below, so that a comparison of the shape of the different distributions in data and MC simulation can be made. The MC expectations provide a fair description of the shapes in data but present harder $E_{\mathrm {T}}^{\mathrm {miss}}$ and leading-jet $p_{\mathrm {T}}$ spectra. This is mainly attributed to an inadequate modelling of the boson $p_{\mathrm {T}}$ distribution in the $W/Z+$jets MC samples.

The data in the control regions and MC-based correction factors, determined from the SHERPA simulation, are used for each of the signal selections (SR1–SR9) to estimate the electroweak background contributions from $W+$jets and $Z(\rightarrow \nu \bar{\nu })+$jets processes. As an example, the $W(\rightarrow \mu \nu )+$jets and $Z(\rightarrow \nu \bar{\nu })+$jets background contributions to a given signal region, $N^{W(\rightarrow \mu \nu )}_\mathrm{{signal}}$ and $N^{Z(\rightarrow \nu \bar{\nu })}_\mathrm{{signal}}$, respectively, are determined using the $W(\rightarrow \mu \nu )+$jets control sample in data according to

1 $$\begin{aligned} N^{W(\rightarrow \mu \nu )}_\mathrm{{signal}}&= \frac{(N^\mathrm{{data}}_{W(\rightarrow \mu \nu ),\mathrm{{control}}} - N^{\mathrm{{non}}-W/Z}_{W(\rightarrow \mu \nu ),\mathrm{{control}}})}{N^{\mathrm{{MC}}}_{W(\rightarrow \mu \nu ),\mathrm{{control}}}} \nonumber \\&\times N^{\mathrm{{MC}} (W(\rightarrow \mu \nu ))}_\mathrm{{signal}} \times \xi _{\ell } \times \xi _\mathrm{{trg}} \times \xi _{\ell }^\mathrm{{veto}} \end{aligned}$$

and

2 $$\begin{aligned} N^{Z(\rightarrow \nu \bar{\nu })}_\mathrm{{signal}}&= \frac{(N^\mathrm{{data}}_{W(\rightarrow \mu \nu ),\mathrm{{control}}} - N^{\mathrm{{non}}-W/Z}_{W(\rightarrow \mu \nu ),\mathrm{{control}}})}{N^{\mathrm{{MC}}}_{W(\rightarrow \mu \nu ),\mathrm{{control}}}} \nonumber \\&\times N^{\mathrm{{MC}} (Z(\rightarrow \nu \bar{\nu }))}_\mathrm{{signal}} \times \xi _{\ell } \times \xi _\mathrm{{trg}}, \end{aligned}$$

where $N^{\mathrm{{MC}} (W(\rightarrow \mu \nu ))}_\mathrm{{signal}}$ and $N^{\mathrm{{MC}} (Z(\rightarrow \nu \bar{\nu }))}_\mathrm{{signal}}$ denote, respectively, the $W(\rightarrow \mu \nu )+$jets and $Z(\rightarrow \nu \bar{\nu })+$jets background predicted by the MC simulation in the signal region, and $N^\mathrm{{data}}_{W(\rightarrow \mu \nu ),\mathrm{{control}}}$, $N^\mathrm{{MC}}_{W(\rightarrow \mu \nu ),\mathrm{{control}}}$, and $N^{\mathrm{{non}}-W/Z}_{W(\rightarrow \mu \nu ),\mathrm{{control}}}$ denote, in the control region, the number of $W(\rightarrow \mu \nu )+$jets candidates in data and $W/Z+$jets MC simulation, and the non-*W* / *Z* background contribution, respectively. The $N^{\mathrm{{non}}-W/Z}_{W(\rightarrow \mu \nu ),\mathrm{{control}}}$ term refers mainly to top-quark and diboson processes, but also includes contributions from multijet processes determined using data. Finally, $\xi _\mathrm{{\ell }}$, $\xi _{\ell }^\mathrm{{veto}}$, and $\xi _\mathrm{{trg}}$ account for possible data–MC differences in the lepton identification, lepton vetoes, and trigger efficiencies, respectively; they typically depart from unity by less than 1 $\%$. The MC-to-data normalization factors (the $(N^\mathrm{{data}}_{W(\rightarrow \mu \nu ),\mathrm{{control}}} - N^{\mathrm{{non}}-W/Z}_{W(\rightarrow \mu \nu ),\mathrm{{control}}})/N^{\mathrm{{MC}}}_{W(\rightarrow \mu \nu ),\mathrm{{control}}}$ term in Eq. (1)) for each process vary between about 0.9 and 0.6 as the required minimum $E_{\mathrm {T}}^{\mathrm {miss}}$ increases from 150 GeV to 700 GeV, and account for the tendency of the MC expectations for $W/Z+$jets processes to exceed the data in the control regions (see, for example, Fig. 2). Similarly, bin-by-bin correction factors are used to correct the shape of the different distributions in the signal regions.

As already mentioned, the different background contributions in the signal regions from $W(\rightarrow \ell \nu )+$jets processes (with $\ell = e, \mu$) are constrained using correction factors obtained from the corresponding control regions. In the case of the $W(\rightarrow \tau \nu )+$jets contributions, the correction factors from the $W(\rightarrow e \nu )+$jets control regions are used. For each of the signal regions, four separate sets of correction factors are considered to constrain the dominant $Z(\rightarrow \nu \bar{\nu })+$jets background contribution, following Eq. (2), as determined separately using $Z/\gamma ^*(\rightarrow \ell ^+ \ell ^-)+$jets and $W(\rightarrow \ell \nu )+$jets control samples. The four resulting $Z(\rightarrow \nu \bar{\nu })+$jets background estimations in each signal region are found to be consistent within uncertainties and are statistically combined using the Best Linear Unbiased Estimate (BLUE) [119] method, which takes into account correlations of systematic uncertainties.

### Multijet background

The multijet background with large $E_{\mathrm {T}}^{\mathrm {miss}}$ mainly originates from the misreconstruction of the energy of a jet in the calorimeter and to a lesser extent from the presence of neutrinos in the final state due to heavy-flavour decays. The multijet background is determined from data, using a *jet smearing* method as described in Ref. [120], which relies on the assumption that the $E_{\mathrm {T}}^{\mathrm {miss}}$ of multijet events is dominated by fluctuations in the detector response to jets measured in the data. For the SR1 and SR2 selections, the multijet background constitutes about $2~\%$ and $0.7~\%$ of the total background, respectively, and is below $0.5~\%$ for the rest of the signal regions with higher $E_{\mathrm {T}}^{\mathrm {miss}}$ thresholds.

### Non-collision background

Detector noise, beam-halo and cosmic muons leading to large energy deposits in the calorimeters represent a significant portion of data acquired by $E_{\mathrm {T}}^{\mathrm {miss}}$ triggers. These non-collision backgrounds resemble the topology of monojet-like final states and require a dedicated strategy to suppress them. The selection described in Sect. 5 is expected to maintain the non-collision background below the percent level. The rate of the fake jets due to cosmic muons surviving the selection criteria, as measured in dedicated cosmic datasets, is found negligible with respect to the rate of data in the monojet-like signal regions. The major source of the non-collision backgrounds is thus beam-halo muons. Since jets due to collisions are expected to be in time with the bunch crossing, an assumption is made that all events containing a leading jet within the out-of-time window are due to beam-induced backgrounds. The characteristic shape of the fake jets due to beam-halo muons is extracted from signal-region events identified as beam-induced backgrounds based on the spatial alignment of the signals in the calorimeter and the muon system [117]. The level of non-collision background in the signal region is extracted as

3 $$\begin{aligned} N_\mathrm {NCB}^\mathrm {SR}=N_{-10<t<-5}^\mathrm {SR}\times \frac{N^\mathrm {NCB}}{N_{-10<t<-5}^\mathrm {NCB}}, \end{aligned}$$

where $N_{-10<t<-5}^\mathrm {SR}$ denotes the number of events in the signal region with a leading jet in the range $-10 {\ \mathrm {ns}} < t < -5 {\ \mathrm {ns}}$, $N_{-10<t<-5}^\mathrm {NCB}$ is the number of identified beam-induced background events there and $N^\mathrm {NCB}$ represents all identified events in the signal region. The results of this study indicate that the non-collision background in the different signal regions is negligible.

## Systematic uncertainties

Several sources of systematic uncertainty are considered in the determination of the background contributions. Uncertainties in the absolute jet energy scale and resolution [111] translate into an uncertainty in the total background which varies from $0.2~\%$ for SR1 and $1~\%$ for SR7 to $3~\%$ for SR9. Uncertainties in the $E_{\mathrm {T}}^{\mathrm {miss}}$ reconstruction introduce an uncertainty in the total background which varies from $0.2~\%$ for SR1 and $0.7~\%$ for SR7 to $1~\%$ for SR9. Uncertainties of the order of 1 $\%$–2 $\%$ in the simulated lepton identification and reconstruction efficiencies, energy/momentum scale and resolution, and a 0.5 $\%$–1 $\%$ uncertainty in the track isolation efficiency translate, altogether, into a $1.4~\%$, $1.5~\%$, and $2~\%$ uncertainty in the total background for the SR1, SR7, and SR9 selections, respectively. Uncertainties of the order of $1~\%$ in the $E_{\mathrm {T}}^{\mathrm {miss}}$ trigger simulation at low $E_{\mathrm {T}}^{\mathrm {miss}}$ and in the efficiency of the lepton triggers used to define the electron and muon control samples translate into uncertainties in the total background of about 0.1 $\%$ for SR1 and become negligible for the rest of the signal regions.

The top-quark-related background contributions, as determined from MC simulations (see Sect. 3), are validated in dedicated validation regions defined similarly to the $W(\rightarrow e \nu )+$jets and $W(\rightarrow \mu \nu )+$jets control regions with $\Delta \phi ({\mathbf {p}}_\mathrm{T}^\mathrm{\ miss}, \mathrm {jet}) > 0.5$ and by requiring the presence of two *b*-tagged jets in the final state with jet $|\eta | < 2.4$. The comparison between data and MC expectations in those validation regions leads to uncertainties in the top-quark background yields which increase from $20~\%$ for SR1 to 100 $\%$ for SR7 and SR9. This translates into uncertainties in the total background expectations which vary from $0.7~\%$ for SR1 and 2.7 $\%$ for SR7 to 4 $\%$ for SR9. Similarly, uncertainties in the simulated diboson background yields include uncertainties in the MC generators and the modelling of parton showers employed, variations in the set of parameters that govern the parton showers and the amount of initial- and final-state soft gluon radiation, and uncertainties due to the choice of renormalization and factorization scales and PDF. This introduces an uncertainty in the diboson background expectation which increases from 20 $\%$ for SR1 to 30 $\%$ for SR7 and 80 $\%$ for SR9. This results in an uncertainty in the total background of 0.7 $\%$, 2.3 $\%$, and 3 $\%$ for the SR1, SR7, and SR9 selections, respectively.

Uncertainties in the $W/Z+$jets modelling include: variations of the renormalization, factorization, and parton-shower matching scales and PDF in the SHERPA $W/Z+$jets background samples; and uncertainties in the parton-shower model considered. In addition, the effect of NLO electroweak corrections on the $W+$jets to $Z+$jets ratio is taken into account [121–123]. Altogether, this translates into an uncertainty in the total background of about 1 $\%$ for SR1 and SR7 and 3 $\%$ for SR9.

Uncertainties in the multijet and $\gamma +$jets background contamination of 100 $\%$ and $50~\%$, respectively, in the $W(\rightarrow e \nu )+$jets control region, propagated to the $Z(\rightarrow \nu \bar{\nu })+$jets background determination in the signal regions, introduce an additional 1 $\%$ uncertainty in the total background for the SR9 selection. The uncertainty in the multijet background contamination in the signal regions leads to a 2 $\%$ and 0.7 $\%$ uncertainty in the total background for the SR1 and SR2 selections, respectively. Finally, the impact of the uncertainty in the total integrated luminosity, which partially cancels in the data-driven determination of the SM background, is negligible.

After including statistical uncertainties in the data and MC expectations in control regions and in the MC expectations in the signal regions, the total background in the signal regions is determined with uncertainties that vary from $2.7~\%$ for SR1 and 6.2 $\%$ for SR7 to $14~\%$ for SR9.

### Signal systematic uncertainties

Several sources of systematic uncertainty in the predicted signal yields are considered for each of the models for new physics. The uncertainties are computed separately for each signal region by varying the model parameters (see Sect. 8).

Experimental uncertainties include: those related to the jet and $E_{\mathrm {T}}^{\mathrm {miss}}$ reconstruction, energy scales and resolutions; those in the proton beam energy, as considered by simulating samples with the lower and upper allowed values given in Ref. [124]; a $1~\%$ uncertainty in the trigger efficiency, affecting only SR1; and the $2.8~\%$ uncertainty in the integrated luminosity. Other uncertainties related to the track veto or the jet quality requirements are negligible ($<$1 $\%$).

Uncertainties affecting the signal acceptance times efficiency $A \times \epsilon$, related to the generation of the signal samples, include: uncertainties in the modelling of the initial- and final-state gluon radiation, as determined using simulated samples with modified parton-shower parameters, by factors of two and one half, that enhance or suppress the parton radiation; uncertainties due to PDF and variations of the $\alpha _\mathrm{s}(m_Z)$ value employed, as computed from the envelope of CT10, MRST2008LO and NNPDF21LO error sets; and the choice of renormalization/factorization scales, and the parton-shower matching scale settings, varied by factors of two and one half.

In addition, theoretical uncertainties in the predicted cross sections, including PDF and renormalization/factorization scale uncertainties, are computed separately for the different models.

## Results and interpretation

**Fig. 6:**
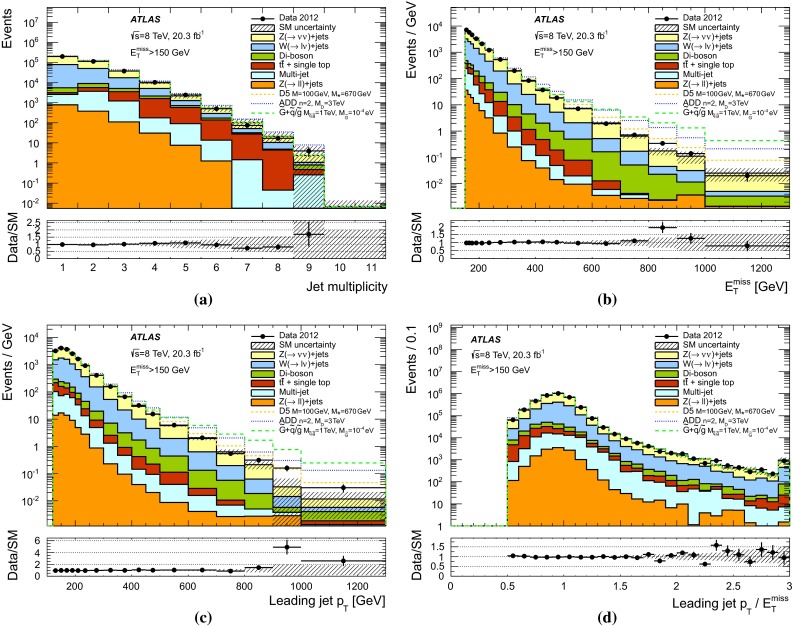
Measured distributions of **a** the jet multiplicity, **b** $E_{\mathrm {T}}^{\mathrm {miss}}$, **c** leading jet $p_{\mathrm {T}}$, and **d** the leading jet $p_{\mathrm {T}}$ to $E_{\mathrm {T}}^{\mathrm {miss}}$ ratio for the SR1 selection compared to the SM expectations. The $Z(\rightarrow \nu \bar{\nu })+$jets contribution is shown as constrained by the $W(\rightarrow \mu \nu )+$jets control sample. Where appropriate, the last bin of the distribution includes overflows. For illustration purposes, the distribution of different ADD, WIMP and GMSB scenarios are included. The *error bands* in the ratios shown in *lower panels* include both the statistical and systematic uncertainties in the background expectations

**Fig. 7:**
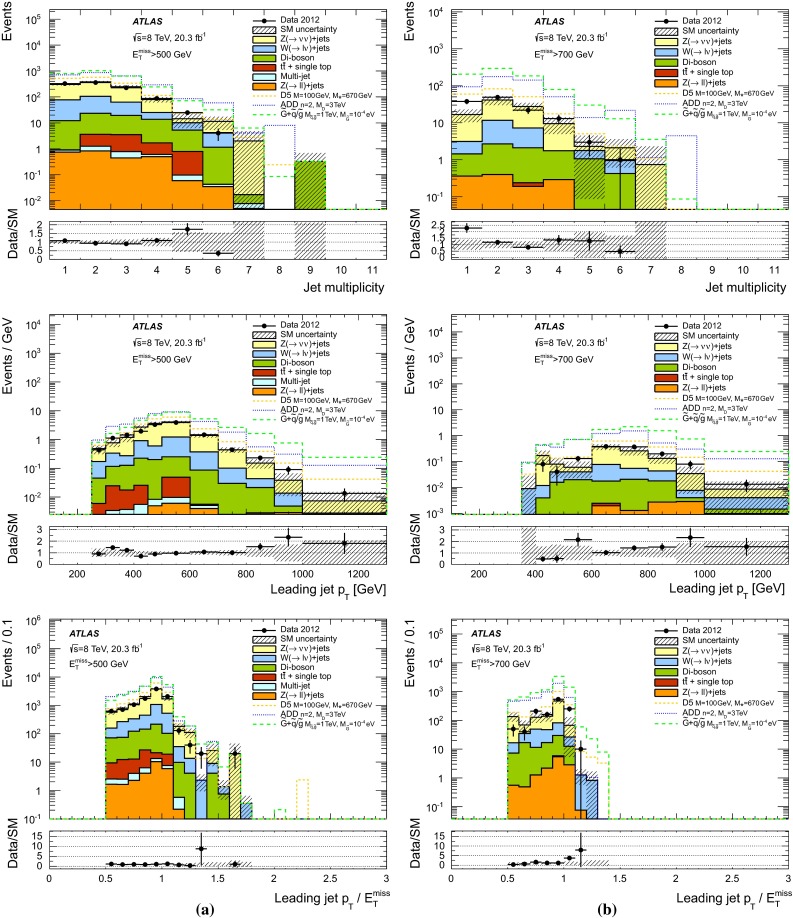
Measured distributions of the jet multiplicity, leading jet $p_{\mathrm {T}}$, and the leading jet $p_{\mathrm {T}}$ to $E_{\mathrm {T}}^{\mathrm {miss}}$ ratio for **a** SR7 and **b** SR9 selections compared to the SM expectations. The $Z(\rightarrow \nu \bar{\nu })+$jets contribution is shown as constrained by the $W(\rightarrow \mu \nu )+$jets control sample. Where appropriate, the last bin of the distribution includes overflows. For illustration purposes, the distribution of different ADD, WIMP and GMSB scenarios are included. The *error bands* in the ratios shown in *lower panels* include both the statistical and systematic uncertainties in the background expectations

The data and the SM expectations in the different signal regions are presented in Tables 4 and 5. In general, good agreement is observed between the data and the SM expectations. The largest difference between the number of events in data and the expectations is observed in the signal region SR9, corresponding to a 1.7$\sigma$ deviation with a *p* value of 0.05, consistent with the background-only hypothesis. Figures 6 and 7 show several measured distributions in data compared to the SM expectations for SR1, and SR7 and SR9, respectively. For illustration purposes, the distributions include the impact of different ADD, WIMP, and GMSB SUSY scenarios.

**Table 4:** Data and SM background expectation in the signal region for the SR1–SR5 selections. For the SM expectations both the statistical and systematic uncertainties are included. In each signal region, the individual uncertainties for the different background processes can be correlated, and do not necessarily add in quadrature to the total background uncertainty

Signal region	SR1	SR2	SR3	SR4	SR5
Observed events	364378	123228	44715	18020	7988
SM expectation	372100 $\pm$ 9900	126000 $\pm$ 2900	45300 $\pm$ 1100	18000 $\pm$ 500	8300 $\pm$ 300
$Z(\rightarrow \nu \bar{\nu })$	217800 $\pm$ 3900	80000 $\pm$ 1700	30000 $\pm$ 800	12800 $\pm$ 410	6000 $\pm$ 240
$W(\rightarrow \tau \nu )$	79300 $\pm$ 3300	24000 $\pm$ 1200	7700 $\pm$ 500	2800 $\pm$ 200	1200 $\pm$ 110
$W(\rightarrow e \nu )$	23500 $\pm$ 1700	7100 $\pm$ 560	2400 $\pm$ 200	880 $\pm$ 80	370 $\pm$ 40
$W(\rightarrow \mu \nu )$	28300 $\pm$ 1600	8200 $\pm$ 500	2500 $\pm$ 200	850 $\pm$ 80	330 $\pm$ 40
$Z/\gamma ^*(\rightarrow \mu ^+\mu ^-)$	530 $\pm$ 220	97 $\pm$ 42	19 $\pm$ 8	7 $\pm$ 3	4 $\pm$ 2
$Z/\gamma ^*(\rightarrow \tau ^+ \tau ^-)$	780 $\pm$ 320	190 $\pm$ 80	45 $\pm$ 19	14 $\pm$ 6	5 $\pm$ 2
$t\bar{t}$, single top	6900 $\pm$ 1400	2300 $\pm$ 500	700 $\pm$ 160	200 $\pm$ 70	80 $\pm$ 40
Dibosons	8000 $\pm$ 1700	3500 $\pm$ 800	1500 $\pm$ 400	690 $\pm$ 200	350 $\pm$ 120
Multijets	6500 $\pm$ 6500	800 $\pm$ 800	200 $\pm$ 200	44 $\pm$ 44	15 $\pm$ 15

**Table 5:** Data and SM background expectation in the signal region for the SR6–SR9 selections. For the SM expectations both the statistical and systematic uncertainties are included. In each signal region, the individual uncertainties for the different background processes can be correlated, and do not necessarily add in quadrature to the total background uncertainty

Signal region	SR6	SR7	SR8	SR9
Observed events	3813	1028	318	126
SM expectation	4000 $\pm$ 160	1030 $\pm$ 60	310 $\pm$ 30	97 $\pm$ 14
$Z(\rightarrow \nu \bar{\nu })$	3000 $\pm$ 150	740 $\pm$ 60	240 $\pm$ 30	71 $\pm$ 13
$W(\rightarrow \tau \nu )$	540 $\pm$ 60	130 $\pm$ 20	34 $\pm$ 8	11 $\pm$ 3
$W(\rightarrow e \nu )$	170 $\pm$ 20	43 $\pm$ 7	9 $\pm$ 3	3 $\pm$ 1
$W(\rightarrow \mu \nu )$	140 $\pm$ 20	35 $\pm$ 6	10 $\pm$ 2	2 $\pm$ 1
$Z/\gamma ^*(\rightarrow \mu ^+\mu ^-)$	3 $\pm$ 1	2 $\pm$ 1	1 $\pm$ 1	1 $\pm$ 1
$Z/\gamma ^*(\rightarrow \tau ^+ \tau ^-)$	2 $\pm$ 1	0 $\pm$ 0	0 $\pm$ 0	0 $\pm$ 0
$t\bar{t}$, single top	30 $\pm$ 20	7 $\pm$ 7	1 $\pm$ 1	0 $\pm$ 0
Dibosons	183 $\pm$ 70	65 $\pm$ 35	23 $\pm$ 16	8 $\pm$ 7
Multijets	6 $\pm$ 6	1 $\pm$ 1	0 $\pm$ 0	0 $\pm$ 0

The agreement between the data and the SM expectations for the total number of events in the different signal regions is translated into model-independent 90 $\%$ and 95 $\%$ confidence level (CL) upper limits on the visible cross section, defined as the production cross section times acceptance times efficiency $\sigma \times A \times \epsilon$, using the $CL_s$ modified frequentist approach [125] and considering the systematic uncertainties in the SM backgrounds and the uncertainty in the integrated luminosity. The results are presented in Table 6. Values of $\sigma \times A \times \epsilon$ above 599 fb–2.9 fb (726 fb–3.4 fb) are excluded at 90 $\%$ CL (95 $\%$ CL) for SR1–SR9 selections, respectively. Typical event selection efficiencies $\epsilon$ varying from $88~\%$ for SR1 and $83~\%$ for SR3 to $82~\%$ for SR7 and $81~\%$ for SR9 are found in simulated $Z(\rightarrow \nu \bar{\nu })+$jets background processes.

**Table 6:** Observed and expected 90 $\%$ CL and 95 $\%$ CL upper limits on the product of cross section, acceptance and efficiency, $\sigma \times A \times \epsilon$, for the SR1–SR9 selections

Upper limits on $\sigma \times A \times \epsilon$ (fb)		
Signal region	90$\%$ CL observed (expected)	95$\%$ CL observed (expected)
SR1	599 (788)	726 (935)
SR2	158 (229)	194 (271)
SR3	74 (89)	90 (106)
SR4	38 (43)	45 (51)
SR5	17 (24)	21 (29)
SR6	10 (14)	12 (17)
SR7	6.0 (6.0)	7.2 (7.2)
SR8	3.2 (3.0)	3.8 (3.6)
SR9	2.9 (1.5)	3.4 (1.8)

### Large extra spatial dimensions

The results are translated into limits on the parameters of the ADD model. The typical $A \times \epsilon$ of the selection criteria vary, as the number of extra dimensions *n* increases from $n=2$ to $n=6$, between 23 $\%$ and 33 $\%$ for SR1 and between $0.3~\%$ and 1.4 $\%$ for SR9, and are approximately independent of $M_D$.

The experimental uncertainties related to the jet and $E_{\mathrm {T}}^{\mathrm {miss}}$ scales and resolutions introduce, when combined, uncertainties in the signal yields which vary between 2 $\%$ and $0.7~\%$ for SR1 and between $8~\%$ and 5 $\%$ for SR9, with increasing *n*. The uncertainties in the proton beam energy result in uncertainties in the signal cross sections which vary between 2 $\%$ and 5 $\%$ with increasing *n*, and uncertainties in the signal acceptance of about 1 $\%$ for SR1 and 3 $\%$–4 $\%$ for SR9. The uncertainties related to the modelling of the initial- and final-state gluon radiation translate into uncertainties in the ADD signal acceptance which vary with increasing *n* between 2 $\%$ and 3 $\%$ in SR1 and between 11 $\%$ and 21 $\%$ in SR9. The uncertainties due to PDF, affecting both the predicted signal cross section and the signal acceptance, result in uncertainties in the signal yields which vary with increasing *n* between 18 $\%$ and 30 $\%$ for SR1 and between 35 $\%$ and 41 $\%$ for SR9. For the SR1 selection, the uncertainty in the signal acceptance itself is about $8~\%$–9 $\%$, and increases to about 30 $\%$ for the SR9 selection. Similarly, the variations of the renormalization and factorization scales introduce a 9 $\%$ to $30~\%$ change in the signal acceptance and a 22 $\%$ to 40 $\%$ uncertainty in the signal yields with increasing *n* and $E_{\mathrm {T}}^{\mathrm {miss}}$ requirements.

**Fig. 8:**
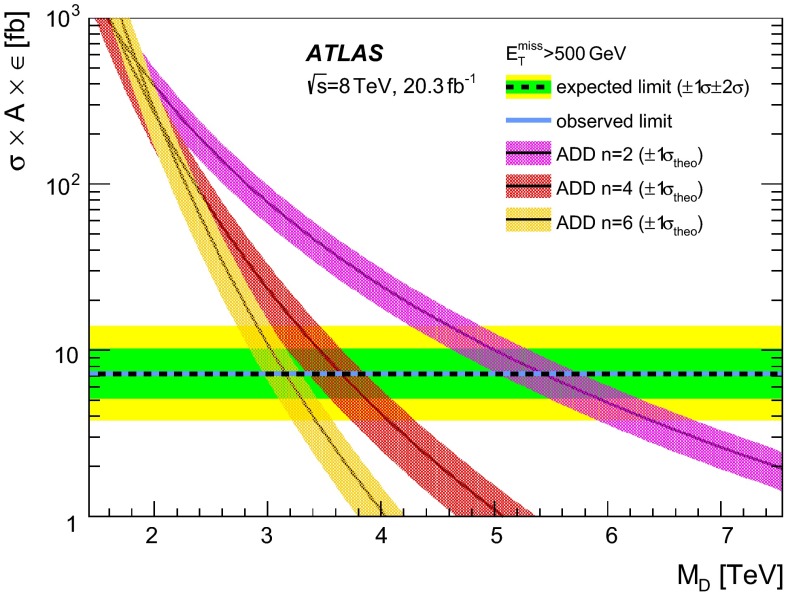
The predicted ADD product of cross section, acceptance and efficiency, $\sigma \times A \times \epsilon$, for the SR7 selection as a function of the fundamental Planck scale in $4+n$ dimensions, $M_D$, for $n=2$, $n=4$, and $n=6$, where *bands* represent the uncertainty in the theory. For comparison, the model-independent observed (*solid line*) and expected (*dashed line*) 95 $\%$ CL limits on $\sigma \times A \times \epsilon$ are shown. The shaded areas around the expected limit indicate the expected $\pm 1\sigma$ and $\pm 2\sigma$ ranges of limits in the absence of a signal

**Fig. 9:**
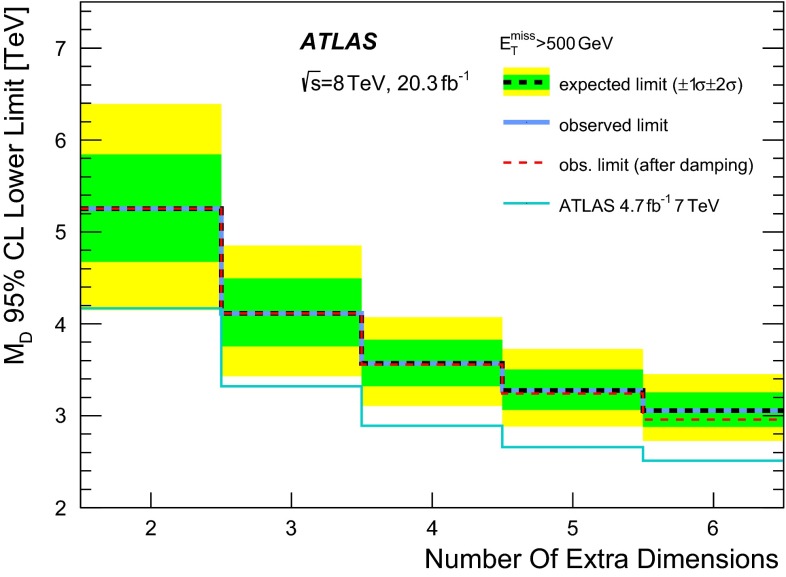
Observed and expected 95 $\%$ CL limit on the fundamental Planck scale in $4+n$ dimensions, $M_D$, as a function of the number of extra dimensions. In the figure the two results overlap. The*shaded areas* around the expected limit indicate the expected $\pm 1\sigma$ and $\pm 2\sigma$ ranges of limits in the absence of a signal. Finally, the *thin dashed line* shows the 95 $\%$ CL observed limits after the suppression of the events with $\hat{s} > M_D^2$ (*damping*) is applied, as described in the body of the text. The results from this analysis are compared to previous results from ATLAS at 7 TeV [12] without any damping applied

The signal region SR7 provides the most stringent expected limits and is used to obtain the final results. Figure 8 shows, for the SR7 selection, the ADD $\sigma \times A \times \epsilon$ as a function of $M_D$ for $n=2$, $n=4$, and $n=6$, calculated at LO. For comparison, the model-independent 95 $\%$ CL limit is shown. Expected and observed 95 $\%$ CL lower limits are set on the value of $M_{D}$ as a function of the number of extra dimensions considered in the ADD model. The $CL_s$ approach is used, including statistical and systematic uncertainties. For the latter, the uncertainties in the signal acceptance times efficiency, the background expectations, and the luminosity are considered, and correlations between systematic uncertainties in signal and background expectations are taken into account. In addition, observed limits are computed taking into account the $\pm 1\sigma$ LO theoretical uncertainty. Values of $M_D$ below 5.25 TeV ($n=2$), 4.11 TeV ($n=3$), 3.57 TeV ($n=4$), 3.27 TeV ($n=5$), and 3.06 TeV ($n=6$) are excluded at 95 $\%$ CL, which extend significantly the exclusion from previous results using 7 TeV data [12]. The observed limits decrease by about $6~\%$–$8~\%$ after considering the $-1\sigma$ uncertainty from PDF and scale variations in the ADD theoretical predictions (see Table 7; Fig. 9).

As discussed in Ref. [12], the analysis partially probes the phase-space region with $\hat{s} > M_D^2$, where $\sqrt{\hat{s}}$ is the centre-of-mass energy of the hard interaction. This challenges the validity of model implementation and the lower bounds on $M_D$, as they depend on the unknown ultraviolet behaviour of the effective theory. For the SR7 selection, the fraction of signal events with $\hat{s} > M_D^2$ is negligible for $n=2$, but increases with increasing *n* from 1 $\%$ for $n=3$ and 6 $\%$ for $n=4$, to about 17 $\%$ for $n=5$ and 42 $\%$ for $n=6$. The observed 95 $\%$ CL limits are recomputed after suppressing, with a weighting factor $M_D^4/\hat{s}^2$, the signal events with $\hat{s} > M_D^2$, here referred to as damping. This results in a decrease of the quoted 95 $\%$ CL on $M_D$ which is negligible for $n=2$ and about 3 $\%$ for $n=6$ (see Fig. 9).

**Table 7:** The $95~\%$ CL observed and expected limits on the fundamental Planck scale in $4+n$ dimensions, $M_D$, as a function of the number of extra dimensions *n* for the SR7 selection and considering LO signal cross sections. The impact of the $\pm 1\sigma$ theoretical uncertainty on the observed limits and the expected $\pm 1\sigma$ range of limits in the absence of a signal are also given. Finally, the $95~\%$ CL observed limits after damping of signal cross section for $\hat{s} > M_D^2$ (see body of the text) are quoted between parentheses

$95~\%$ CL limits on $M_D$ (TeV)						
*n* extra dimensions	$95~\%$ CL observed limit			$95~\%$ CL expected limit		
	$+1\sigma$ (theory)	Nominal (nominal after damping)	$-1\sigma$ (theory)	$+1\sigma$	Nominal	$-1\sigma$
2	$+$0.31	5.25 (5.25)	$-$0.38	$-$0.59	5.25	$+$0.58
3	$+$0.25	4.11 (4.11)	$-$0.33	$-$0.38	4.11	$+$0.36
4	$+$0.20	3.57 (3.56)	$-$0.29	$-$0.26	3.57	$+$0.25
5	$+$0.17	3.27 (3.24)	$-$0.25	$-$0.23	3.27	$+$0.21
6	$+$0.13	3.06 (2.96)	$-$0.19	$-$0.20	3.06	$+$0.18

### Weakly interacting massive particles

In the following, the results are converted into limits on the pair production of WIMPs. As illustrated in Fig. 1, this is done both in the EFT framework and in a simplified model where the WIMP pair couples to Standard Model quarks via a $Z^\prime$ boson.

For each EFT operator defined in Table 1, the limits on ${M}_\star$ are extracted from those signal regions that exhibit the best expected sensitivity: these are SR4 for C1, SR7 for D1, D5, D8, and SR9 for C5, D9, D11. These are translated into corresponding 95 % CL limits on the suppression scale ${M}_\star$ as a function of $m_\chi$.To derive these lower limits on ${M}_\star$, the same $CL_s$ approach as in the case of the ADD LED model is used. The uncertainties in the WIMP signal acceptance include: a $3~\%$ uncertainty from the uncertainty in the beam energy; a $3~\%$ uncertainty from the variation of the renormalization and factorization scales and a $5~\%$ uncertainty from the variation of the parton-shower matching scale; a $1~\%$ to $10~\%$ uncertainty from uncertainties in jet and $E_{\mathrm {T}}^{\mathrm {miss}}$ energy scale and resolution; and a $5~\%$ to $29~\%$ uncertainty due to PDF, depending on the operator and WIMP mass.

**Fig. 10:**
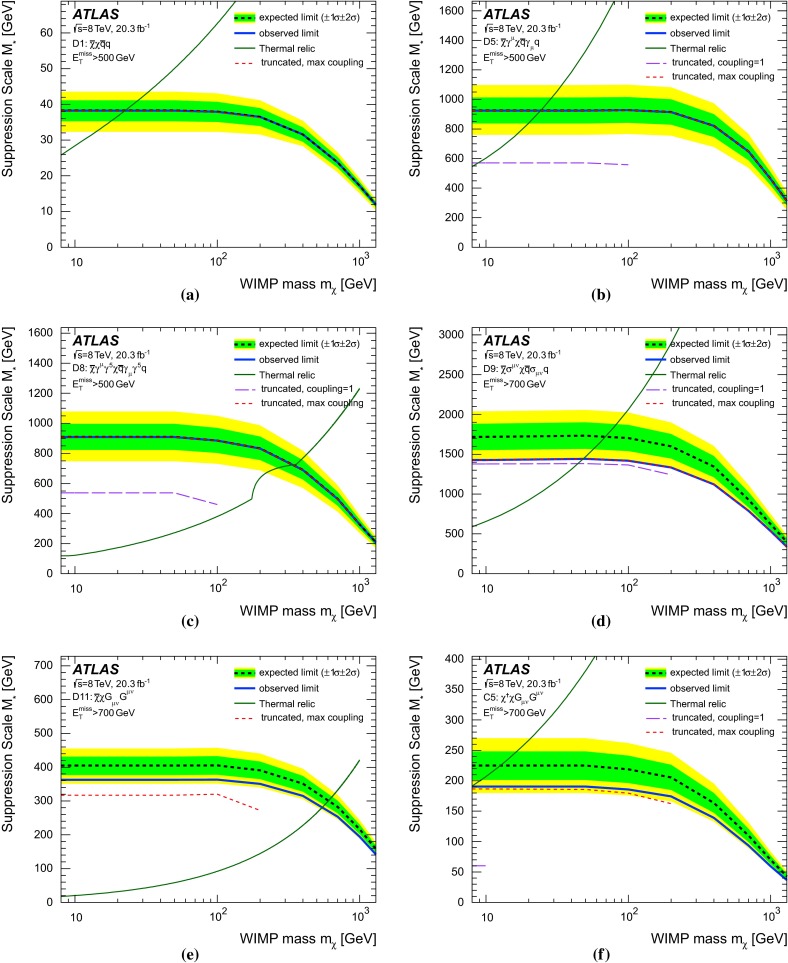
Lower limits at 95 % CL on the suppression scale $M_*$ are shown as a function of the WIMP mass $m_\chi$ for **a** D1, **b** D5, **c** D8, **d** D9, **e** D11 and **f** C5 operators, in each case for the most sensitive SR (SR7 for D1, D5, D8, SR9 for D9, D11 and C5). The expected and observed limits are shown as *dashed black* and *solid blue lines*, respectively. The *rising green lines* are the ${M}_\star$ values at which WIMPs of the given mass result in the relic density as measured by WMAP [27], assuming annihilation in the early universe proceeded exclusively via the given operator. The *purple long-dashed line* is the 95 $\%$ CL observed limit on ${M}_\star$ imposing a validity criterion with a coupling strength of 1, the *red dashed thin lines* are those for the maximum physical coupling strength (see Appendix A for further details)

Similarly, the uncertainties in the signal cross section are: a $2~\%$ to $17~\%$ ($40~\%$ to $46~\%$) uncertainty due to the variation of the renormalization and factorization scales in D1, D5 and D9 (C5 and D11) operators; and a $19~\%$ to $70~\%$ ($5~\%$ to $36~\%$) uncertainty due to the PDF for C5, D11 and D1 (D5 and D9) operators, with increasing WIMP mass. These theoretical cross-section uncertainties are not considered when deriving limits and are not displayed in the plots. A $2~\%$ to $9~\%$ uncertainty in the cross section, due to the beam energy uncertainty, is taken into account.

The ${M}_\star$ limits for five of the operators are shown in Fig. 10 down to WIMP masses of 10 $\,\hbox {GeV}$, and could be extrapolated even to smaller $m_\chi$ values since there is a negligible change in the cross section or the kinematic distributions at the LHC for such low-mass WIMPs. The 1$\sigma$ and 2$\sigma$ error bands around the expected limit are due to the acceptance uncertainties (experimental and theoretical). The effect of the beam-energy uncertainty on the observed limit is negligible and is not shown.

Various authors have investigated the kinematic regions in which the effective field theory approach for WIMP pair production breaks down [43–46]. The problem is addressed in detail in Appendix A, where the region of validity of this approach is probed for various assumptions about the underlying unknown new physics. Here, the EFT framework is used as a benchmark to convert the measurement, and in the absence of any deviation from the SM backgrounds, to a limit on the pair production of DM (with the caveat of not complete validity in the full kinematic phase space). These are the central values of the observed and expected limits in Fig. 10. A basic demonstration of the validity issue is also included in the figure. This is done by relating the suppression scale ${M}_\star$ to the mass of the new particle mediating the interaction, ${M}_\mathrm {med}$, and the coupling constants of the interaction, $g_i$ by

$$\begin{aligned} {M}_\mathrm {med}{} = f(g_i, {M}_\star {}). \end{aligned}$$

For such a relation, an assumption has to be made about the interaction structure connecting the initial state to the final state via the mediator particle. The simplest interaction structures are assumed in all cases. The form of the function *f* connecting ${M}_\mathrm {med}$ and ${M}_\star$ depends then on the operator (see Appendix A). For a given operator, one possible validity criterion is that the momentum transferred in the hard interaction, $Q_\mathrm{tr}$, is below the mediator particle mass: $Q_\mathrm{tr} < {M}_\mathrm {med}{}$. According to this criterion, events are omitted where the interaction energy scale exceeds the mediator particle mass. This depends on the values adopted for the couplings. Two values (one and the maximum possible value for the interaction to remain perturbative) are used. After reducing the signal cross section to the fraction of remaining events, the mass suppression scale ${M}_\star$ can be rederived yielding potentially two additional expected truncated limit lines in Fig. 10. The truncated limits fulfil the respective validity criteria wherever the lines are drawn in the figure. For D9 for example, the maximum couplings criterion is fulfilled for all WIMP masses, the coupling equal to one criterion is fulfilled for WIMP masses up to 200 GeV. For C5 on the other hand, the validity criterion for a coupling value of one is violated over almost the whole WIMP mass range, and a truncated limit line is only drawn up to a WIMP mass of 10 GeV.

Figure 10 also includes thermal relic lines (taken from Ref. [41]) that correspond to a coupling, set by $M_*$, of WIMPs to quarks or gluons such that WIMPs have the correct relic abundance as measured by the WMAP satellite, in the absence of any interaction other than the one considered. The thermal relic line for D8 has a bump feature at the top-quark mass where the annihilation channel to top quarks opens. Under the assumption that DM is entirely composed of thermal relics, the limits on $M_*$ which are above the value required for the thermal relic density exclude the case where DM annihilates exclusively to SM particles via the corresponding operator. Should thermal relic WIMPs exist in these regions (above the thermal relic line), there would have to be other annihilation channels or annihilation via other operators in order to be consistent with the WMAP measurements.

**Fig. 11:**
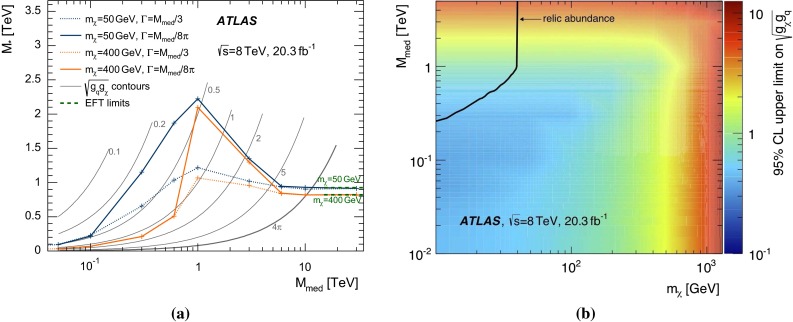
**a** Observed 95 $\%$ CL limits on the suppression scale $M_*$ as a function of the mediator mass ${M}_\mathrm {med}$, assuming a $Z^\prime$-like boson in a simplified model and a DM mass of 50 GeV and 400 GeV. The width of the mediator is varied between ${M}_\mathrm {med}/3$ and ${M}_\mathrm {med}/8\pi$. The corresponding limits from EFT models are shown as *dashed lines*; *contour lines* indicating a range of values of the product of the coupling constants ($\sqrt{\mathrm {g}_q\, \mathrm {g}_\chi }$) are also shown. **b** Observed 95 % CL upper limits on the product of couplings of the simplified model vertex in the plane of mediator and WIMP mass (${M}_\mathrm {med}$ versus $m_\chi$). Values leading to the correct relic abundance [27] are shown by the *black solid line*

Another way to avoid the validity issues discussed above is to use a simplified model to explicitly parameterize the interaction of quarks or gluons with WIMP pairs via generic interactions with real mediator particles. With this approach, the coupling of pairs of Dirac fermion WIMPs to quarks via a vector mediator particle (such as a $Z^\prime$ boson, corresponding to the operator D5) of a given mass and width (${M}_\mathrm {med}$ and $\Gamma$, respectively) is probed. Given the cross-section limit and using simulations at fixed values of ${M}_\mathrm {med}$ and $\Gamma$, the product of the coupling constants of the $Z^\prime$ boson to quarks and WIMPs, $\sqrt{\mathrm {g}_q\, \mathrm {g}_\chi }$, can be constrained. This constraint corresponds to one value in the ${M}_\star$–${M}_\mathrm {med}$ plane as shown in Fig. 11a, since the mass suppression scale can be calculated exactly in this model, ${M}_\star {} = {{M}_\mathrm {med}{}}/{\sqrt{\mathrm {g}_q\, \mathrm {g}_\chi }}$. The figure demonstrates how, for a given mediator particle mass and two values of the width $\Gamma$, the real value of the mass suppression scale would compare to the ${M}_\star$ value derived assuming a contact interaction (shown as dashed lines in the figure). This contact interaction regime is reached for ${M}_\mathrm {med}$ values larger than 5 TeV in the figure. In the intermediate range (700 GeV $< {M}_\mathrm {med}{} < 5$ TeV), the mediator would be produced resonantly and the actual ${M}_\star$ value is higher than in the contact interaction regime. In this case the contact interaction limits would be pessimistic: they would underestimate the actual values. Finally, the small mediator mass regime below 700 GeV has very small ${M}_\star$ limits because the WIMP would be heavier than the mediator, and WIMP pair production via this mediator would thus be kinematically suppressed. In this region, the contact interaction limits would be optimistic and overestimate the actual ${M}_\star$ values.

In Fig. 11b the observed 95 % CL upper limits on the product of couplings of the simplified model vertex are shown in the plane of mediator and WIMP mass (${M}_\mathrm {med}$ versus $m_\chi$). Within this model, the regions to the left of the relic density line lead to values of the relic density larger than measured and are excluded.

**Fig. 12:**
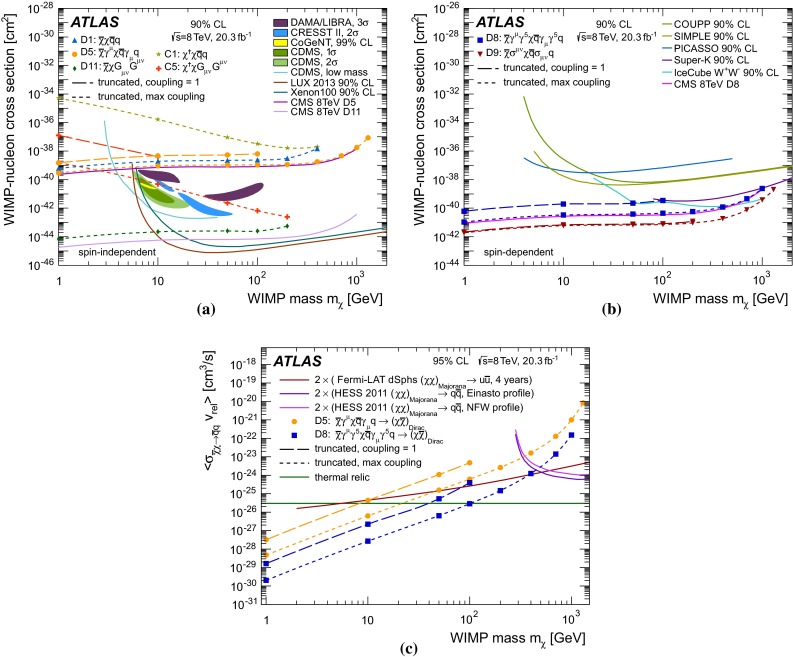
Inferred 90 % CL limits on **a** the spin-independent and **b** spin-dependent WIMP–nucleon scattering cross section as a function of DM mass $m_\chi$ for different operators (see Sect. 1). Results from direct-detection experiments for the spin-independent [128–134] and spin-dependent [135–139] cross section, and the CMS (untruncated) results [14] are shown for comparison. **c** The inferred 95 % CL limits on the DM annihilation rate as a function of DM mass. The annihilation rate is defined as the product of cross section $\sigma$ and relative velocity *v*, averaged over the DM velocity distribution $(\langle \sigma \ v\rangle )$. Results from gamma-ray telescopes [126, 127] are also shown, along with the thermal relic density annihilation rate [26, 27]

In the effective operator approach, the bounds on $M_*$ for a given $m_\chi$ (see Fig. 10) can be converted to bounds on WIMP–nucleon scattering cross sections, which are probed by direct DM detection experiments. These bounds describe scattering of WIMPs from nucleons at a very low momentum transfer of the order of a keV. Depending on the type of interaction, contributions to spin-dependent or spin-independent WIMP–nucleon interactions are expected. As in Ref. [12], the limits are converted here to bounds on the WIMP–nucleon scattering cross sections and the results are displayed in Fig. 12. Under the assumptions made in the EFT approach, the ATLAS DM limits are particularly relevant in the low DM mass region, and remain important over the full $m_\chi$ range covered. The spin-dependent limits in Fig. 12 are based on D8 and D9, where for D8 the $M_*$ limits are calculated using the D5 acceptances (as they are identical) together with D8 production cross sections. Both the D8 and D9 cross-section limits are significantly stronger than those from direct-detection experiments.

The DM limits are shown as upper limits on the WIMP annihilation rate, calculated using the same approach as in Ref. [12], in the bottom panel of Fig. 12. The operators describing the vector and axial-vector annihilations of WIMPs to the four light-quark flavours are shown in this plot. For comparison, limits on the annihilation to $u\bar{u}$ and $q \bar{q}$ from galactic high-energy gamma-ray observations by the Fermi-LAT [126] and H.E.S.S. [127] telescopes are also shown. The gamma-ray limits are for Majorana fermions and are therefore scaled up by a factor of two for comparison with the ATLAS limits for Dirac fermions (see Ref. [12] and references therein for further discussions and explanations). The annihilation rate that corresponds to the thermal relic density measured by WMAP [27] and PLANCK [26] satellites is also shown for comparison in the figure.

Finally, Fig. 12 also demonstrates the impact of the EFT validity and the truncation procedure explained above on the quoted upper limits for the WIMP–nucleon scattering and WIMP annihilation cross sections. The effect depends strongly on the operator and the values for the couplings considered. In general, the limits remain valid for WIMP masses up to *O*(100) GeV. The variation of the coupling strengths considered leads to changes in the quoted cross-section limits of up to one order of magnitude.

### Associated production of a light gravitino and a squark or gluino

The results are also expressed in terms of 95 $\%$ CL limits on the cross section for the associated production of a gravitino and a gluino or a squark. As already discussed, a SUSY simplified model is used in which the gluino and squark decays lead to a gravitino and a gluon or a quark, respectively, producing a monojet-like signature in the final state. Squark and gluino masses up to 2.6 TeV are considered. The acceptance and efficiency $A \times \epsilon$ for the SUSY signal depends on the mass of the squark or gluino in the final state and also on the relation between squark and gluino masses. As an example, in the case of squarks and gluinos degenerate in mass $(m_{\tilde{g}}= m_{\tilde{q}})$, the signal $A \times \epsilon$ for the SR7 (SR9) selection criteria is in the range 25 $\%$–45 $\%$ (10 $\%$–35 $\%$) for squark and gluino masses of about 1–2 TeV.

The systematic uncertainties in the SUSY signal yields are determined as in the case of the ADD and WIMP models. The uncertainties related to the jet and $E_{\mathrm {T}}^{\mathrm {miss}}$ scales and resolutions introduce uncertainties in the signal yields which vary between 2 $\%$ and 16 $\%$ for different selections and squark and gluino masses. The uncertainties in the proton beam energy introduce uncertainties in the signal yields which vary between 2 $\%$ and 6 $\%$ with increasing squark and gluino masses. The uncertainties related to the modelling of initial- and final-state gluon radiation translate into a 10 $\%$ to 15 $\%$ uncertainty in the signal yields, depending on the selection and the squark and gluino masses. The uncertainties due to PDF result in uncertainties in the signal yields which vary between 5 $\%$ and 60 $\%$ for squark and gluino masses increasing from 50 GeV and 2.6 TeV. Finally, the variations of the renormalization and factorization scales introduce a 15 $\%$ to 35 $\%$ uncertainty in the signal yields with increasing squark and gluino masses.

**Fig. 13:**
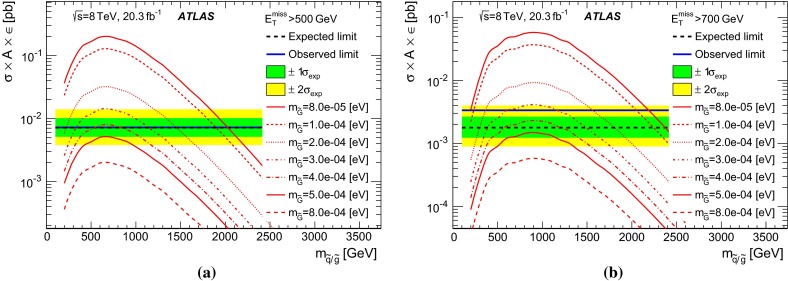
Cross section times acceptance times efficiency $\sigma \times A \times \epsilon$ for gravitino$+$squark/gluino production as a function of the squark/gluino mass $m_{\tilde{q}/\tilde{g}}$ in the case of degenerate squarks and gluinos and different gravitino masses for **a** SR7 and **b** SR9, compared with the corresponding model-independent limits

**Fig. 14:**
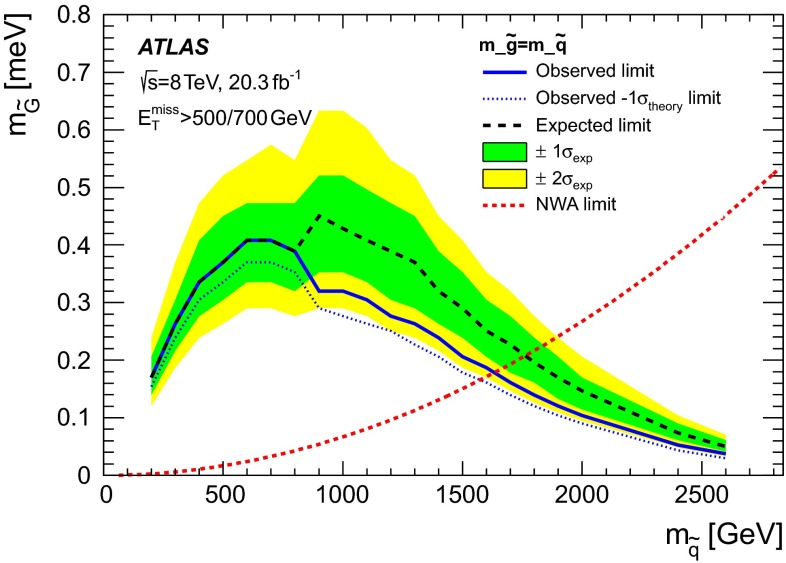
Observed (*solid line*) and expected (*dashed line*) 95 $\%$ CL lower limits on the gravitino mass $m_{\tilde{G}}$ as a function of the squark mass $m_{\tilde{q}}$ for degenerate squark/gluino masses. The corresponding *dotted line* indicates the impact on the observed limit of the $-1\sigma$ LO theoretical uncertainty. The *shaded bands* around the expected limit indicate the expected $\pm 1\sigma$ and $\pm 2\sigma$ ranges of limits in the absence of a signal. The region above the *red dotted line* defines the validity of the narrow-width approximation (NWA) for which the decay width is smaller than 25 $\%$ of the squark/gluino mass

**Fig. 15:**
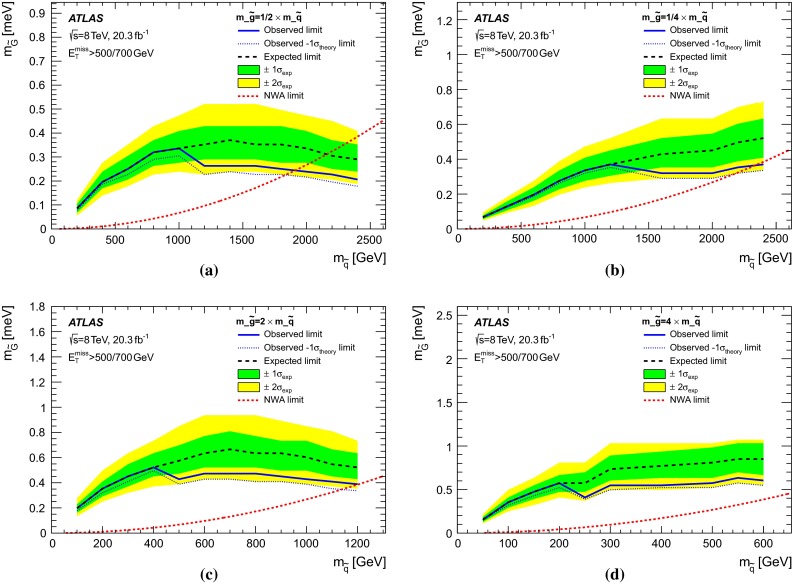
Observed (*solid line*) and expected (*dashed line*) 95 % CL lower limits on the gravitino mass $m_{\tilde{G}}$ as a function of the squark mass $m_{\tilde{q}}$ for non-degenerate squark/gluino masses and different squark/gluino mass configurations. The *dotted line* indicates the impact on the observed limit of the $-1\sigma$ LO theoretical uncertainty. The *shaded bands* around the expected limit indicate the expected $\pm 1\sigma$ and $\pm 2\sigma$ ranges of limits in the absence of a signal. The region above the *red dotted line* defines the validity of the narrow-width approximation (NWA) for which the decay width is smaller than 25 $\%$ of the squark/gluino mass

Figure 13 presents, for the SR7 and SR9 selections and in the case of degenerate squarks and gluinos, $\sigma \times A \times \epsilon$ as a function of the squark/gluino mass for different gravitino masses. For comparison, the model-independent 95 $\%$ CL limits are shown. For each SUSY point considered in the gravitino–squark/gluino mass plane, observed and expected 95 $\%$ CL limits are computed using the same procedure as in the case of the ADD and WIMPs models. This is done separately for the different selections, and the one with the most stringent expected limit is adopted as the nominal result. In the region with squark/gluino masses below 800 GeV, SR7 provides the best sensitivity while SR9 provides the most stringent expected limits for heavier squark/gluino masses. Figure 14 presents the final results. Gravitino masses below $3.5 \times 10^{-4}$ eV, $3 \times 10^{-4}$ eV, and $2 \times 10^{-4}$ eV are excluded at 95 $\%$ CL for squark/gluino masses of 500 GeV, 1 TeV, and 1.5 TeV, respectively. The observed limits decrease by about $9~\%$–$13~\%$ after considering the $-1\sigma$ uncertainty from PDF and scale variations in the theoretical predictions. These results are significantly better than previous results at LEP [55] and the Tevatron [16], and constitute the most stringent bounds on the gravitino mass to date. For very high squark/gluino masses, the partial width for the gluino or squark to decay into a gravitino and a parton becomes more than 25 $\%$ of its mass and the narrow-width approximation employed is not valid any more. In this case, other decay channels for the gluino and squarks should be considered, leading to a different final state. The corresponding region of validity of this approximation is indicated in the figure. Finally, limits on the gravitino mass are also computed in the case of non-degenerate squarks and gluinos (see Fig. 15). Scenarios with $m_{\tilde{g}}= 4 \times m_{\tilde{q}}$, $m_{\tilde{g}}= 2 \times m_{\tilde{q}}$, $m_{\tilde{g}}= 1/2 \times m_{\tilde{q}}$, and $m_{\tilde{g}}= 1/4 \times m_{\tilde{q}}$ have been considered. In this case, 95 $\%$ CL lower bounds on the gravitino mass in the range between $1 \times 10^{-4}$ eV and $5 \times 10^{-4}$ eV are set depending on the squark and gluino masses.

### Invisibly decaying Higgs-like boson

The results are translated into 95 $\%$ CL limits on the production cross section times the branching ratio for a Higgs boson decaying into invisible particles as a function of the boson mass. The SR3 selection provides the best sensitivity to the signal and it is used for the final results. The $A \times \epsilon$ of the selection criteria depends on the production mechanism and the boson mass considered. In the case of the $gg \rightarrow H$ process, the $A \times \epsilon$ varies between 0.1 $\%$ and 0.7 $\%$ with increasing boson mass from 115 GeV to 300 GeV. It varies between 1 $\%$ and 2 $\%$ for the $VV \rightarrow H$ production process, and varies between 1 $\%$ and 12 $\%$ in the *VH* case. The $gg \rightarrow H$ process dominates the signal yield and constitutes more than 52 $\%$ and 67 $\%$ of the boson signal for a boson mass of 125 GeV and 300 GeV, respectively.

The uncertainties related to the jet and $E_{\mathrm {T}}^{\mathrm {miss}}$ scales and resolutions introduce uncertainties in the signal yields for the SR3 signal region which vary between 10 $\%$ and 6 $\%$ for the $gg \rightarrow H$ and $VV \rightarrow H$ processes as the boson mass increases. Similarly, in the case of *VH* production processes, these uncertainties vary between 8 $\%$ and 4 $\%$ with increasing mass. The variations of the renormalization and factorization scales introduce a 8 $\%$ to 6 $\%$, 0.2 $\%$ to 0.8 $\%$, and 1 $\%$ to 3 $\%$ uncertainty in the boson signal yields for $gg \rightarrow H$, $VV \rightarrow H$, and *VH* processes, respectively, as the mass increases. The uncertainties due to PDF result in uncertainties in the signal yields which vary between 7 $\%$ and 8 $\%$, 2 $\%$ and 4 $\%$, and 2 $\%$ and 4 $\%$ for $gg \rightarrow H$, $VV \rightarrow H$, and *VH* processes, respectively. The uncertainty in the parton shower modelling results in a 7 $\%$ uncertainty in the signal yields for the different channels.

**Fig. 16:**
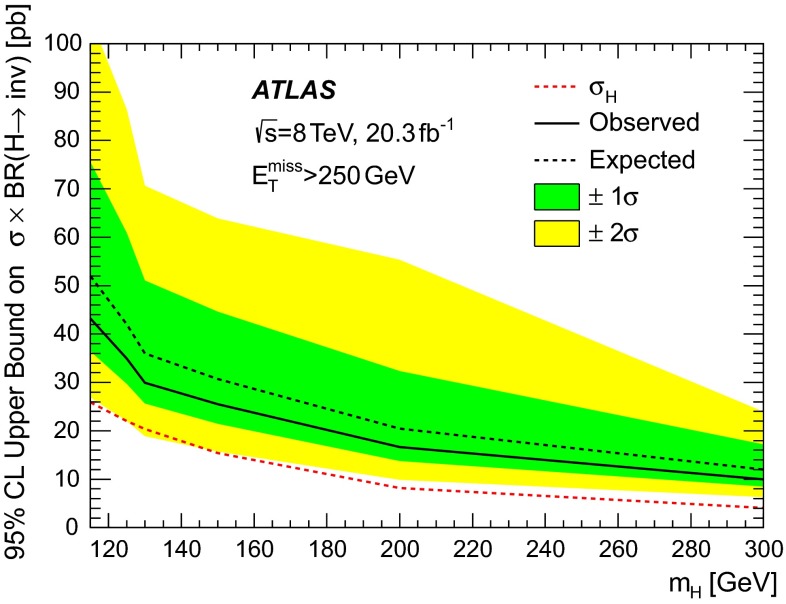
The observed (*solid line*) and expected (*dashed line*) 95 $\%$ CL upper limit on $\sigma \times \mathrm{{BR}}(H \rightarrow \mathrm{{invisible}})$ as a function of the boson mass $m_{H}$. The *shaded areas* around the expected limit indicate the expected $\pm 1\sigma$ and $\pm 2\sigma$ ranges of limits in the absence of a signal. The expectation for a Higgs boson with $\mathrm{{BR}}(H \rightarrow \mathrm{{invisible}}) = 1$, $\sigma _{H}$, is also shown

Figure 16 shows the observed and expected 95 $\%$ CL limits on the cross section times branching ratio $\sigma \times \mathrm{{BR}}(H \rightarrow \mathrm{{invisible}})$ as a function of the boson mass, for masses in the range between 115 GeV and 300 GeV. Values for $\sigma \times \mathrm{{BR}}(H \rightarrow \mathrm{{invisible}})$ above 44 pb for $m_H = 115$ GeV and 10 pb for $m_H = 300$ GeV are excluded. This is compared with the expectation for a Higgs boson with $\mathrm{{BR}}(H \rightarrow \mathrm{{invisible}}) = 1$. For a mass of 125 GeV, values for $\sigma \times \mathrm{{BR}}(H \rightarrow \mathrm{{invisible}})$ 1.59 times larger than the SM predictions are excluded at 95 $\%$ CL, with an expected sensitivity of 1.91 times the SM predictions. This indicates that, for a mass of 125 GeV, this result is less sensitive than that in Ref.[59] using $ZH (Z \rightarrow \ell ^+ \ell ^-)$ final states, and it does not yet have the sensitivity to probe the SM Higgs boson couplings to invisible particles. Nevertheless, for a Higgs boson mass above 200 GeV this analysis gives comparable results.

## Conclusions

In summary, results are reported from a search for new phenomena in events with an energetic jet and large missing transverse momentum in proton–proton collisions at $\sqrt{s}=8$ TeV at the LHC, based on ATLAS data corresponding to an integrated luminosity of 20.3 fb${}^{-1}$. The measurements are in agreement with the SM expectations. The results are translated into model-independent 90 $\%$ and 95 $\%$ confidence-level upper limits on $\sigma \times A \times \epsilon$ in the range 599–2.9 fb and 726–3.4 fb, respectively, depending on the selection criteria considered. The results are presented in terms of limits on the fundamental Planck scale, $M_D$, versus the number of extra spatial dimensions in the ADD LED model, upper limits on the spin-independent and spin-dependent contributions to the WIMP–nucleon elastic cross section as a function of the WIMP mass, and upper limits on the production of very light gravitinos in gauge-mediated supersymmetry. In addition, the results are interpreted in terms of the production of an invisibly decaying Higgs boson for which the analysis shows a limited sensitivity.

## Figures and Tables

**Fig. 17:**
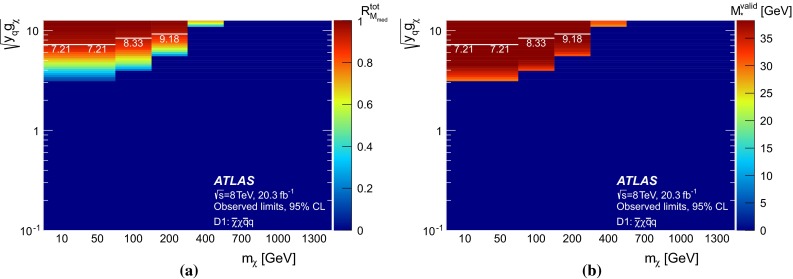
**a** The fraction of valid events and **b** truncated limits for D1 at 95 % CL as a function of the WIMP mass $m_\chi$ and couplings. The *white numbers* correspond to the minimum coupling value for which $M_{*}^{\mathrm {valid}}/M_{*}^{\mathrm {exp}} > 99\%$. The upper perturbative coupling limit for D1 is $4\pi$

**Fig. 18:**
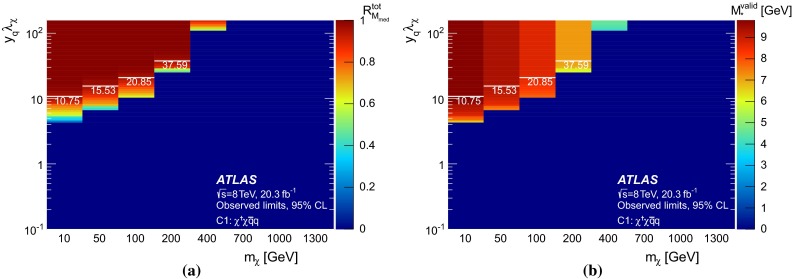
**a** The fraction of valid events and **b** truncated limits for C1 at 95 % CL as a function of the WIMP mass $m_\chi$ and couplings. The *white numbers* correspond to the minimum coupling value for which $M_{*}^{\mathrm {valid}}/M_{*}^{\mathrm {exp}} > 99\%$. The upper perturbative coupling limit for C1 is $(4\pi )^2\zeta _\lambda {}$, where $\zeta _\lambda$ is taken to be 1

**Fig. 19:**
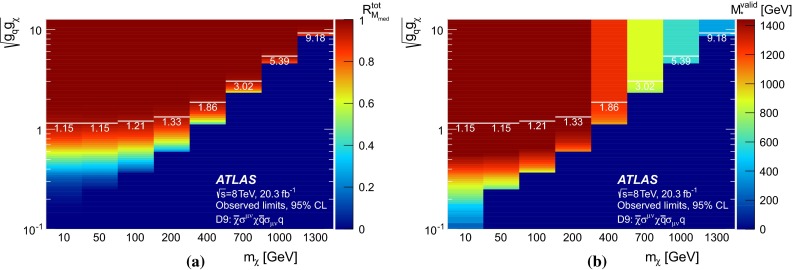
**a** The fraction of valid events and **b** truncated limits for D9 at 95 % CL as a function of the WIMP mass $m_\chi$ and couplings. The *white numbers* correspond to the minimum coupling value for which $M_{*}^{\mathrm {valid}}/M_{*}^{\mathrm {exp}} > 99\%$. The upper perturbative coupling limit for D9 is $4\pi$

**Fig. 20:**
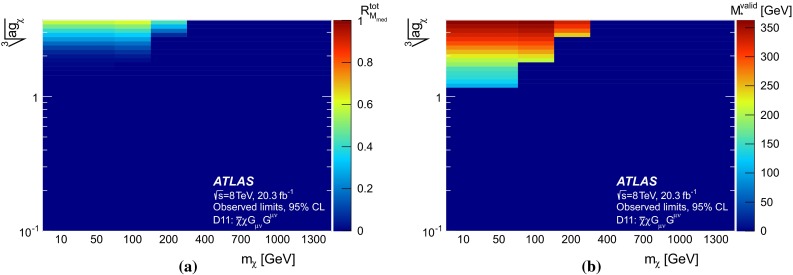
**a** The fraction of valid events and **b** truncated limits for D11 at 95 % CL as a function of the WIMP mass $m_\chi$ and couplings. The *white numbers* correspond to the minimum coupling value for which $M_{*}^{\mathrm {valid}}/M_{*}^{\mathrm {exp}} > 99\%$. The upper perturbative coupling limit for D11 is $\root 3 \of {16\pi }$

**Fig. 21:**
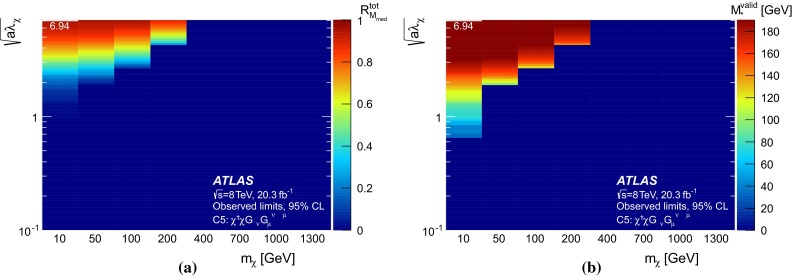
**a** The fraction of valid events and **b** truncated limits for C5 at 95 % CL as a function of the WIMP mass $m_\chi$ and couplings. The *white numbers* correspond to the minimum coupling value for which $M_{*}^{\mathrm {valid}}/M_{*}^{\mathrm {exp}} > 99\%$. The upper perturbative coupling limit for C5 is $4\sqrt{\pi \zeta _\lambda {}}$, where $\zeta _\lambda$ is taken to be 1

## Appendix A: On the validity of the effective field theory used to describe dark-matter pair production

### Appendix A.1: Introduction

The effective field theories (EFTs) used here are based on the assumption that a new mediator particle couples Standard Model particles to pairs of DM particles and that the mediator particle mass is considerably larger than the energy scale of the interaction. In such a case the mediator cannot be produced directly in LHC collisions and can be integrated out with an EFT formalism. This heavy-mediator assumption is indeed justifiable in *direct detection* WIMP scattering experiments due to the very low momentum exchange typically of order keV in the scattering interactions. This assumption is not always correct at the LHC, where the momentum transfer reaches the $\,\hbox {TeV}$ scale [43–46].

A minimal condition for the EFT to be valid is that the momentum transferred in the hard interaction at the LHC does not exceed the mediator particle mass, thus ensuring that the mediator cannot be produced directly: $Q_\mathrm{{tr}}{} < {M}_\mathrm {med}{}$. To probe this validity, further assumptions have to be made about the actual form of the interaction vertex, and thereby about the (unknown) interaction structure itself, connecting quarks or gluons to WIMPs.

The simplest of such assumptions are made below for all the operators used here to derive expressions for ${M}_\mathrm {med}$, ${M}_\star$, and the interaction coupling constants, to probe the minimal validity criterion.

### Appendix A.2: Connecting ${M}_\star$ to ${M}_\mathrm {med}$

The simplest interaction structure for the operators D5, D8, and D9 is an *s*-channel diagram, where the mediator particle couples to the initial-state quarks and the final-state WIMPs. This interaction is described by three parameters, the mediator mass ${M}_\mathrm {med}$, the quark–mediator coupling constant $g_\mathrm {q}$, and the mediator–WIMP coupling constant $g_\chi$. The relation of these parameters to ${M}_\star$ is

$$\begin{aligned} {M}_\mathrm {med}{} = \sqrt{g_\mathrm {q}{}g_\chi {}}{}{M}_\star {}. \end{aligned}$$

The theory is no longer in the perturbative regime if the couplings are outside of the range $0<\sqrt{g_\mathrm {q}{}g_\chi {}}{}<4\pi$.

The simplest *s*-channel diagram for the operators D1 and C1 involves the exchange of a scalar mediator particle where the quark–mediator coupling constant is a Yukawa coupling $y_\mathrm {q}$. In this case, the mediator particle masses can be expressed as:

$$\begin{aligned}&\mathbf{D1}&\mathbf{C1}\\ \frac{m_q}{{M}_\star ^3}&= \frac{y_\mathrm {q}{}g_\chi {}}{{M}_\mathrm {med}^2}&\frac{m_q}{{M}_\star ^2}&= \frac{y_\mathrm {q}{}\lambda _\chi {}\nu _\lambda {}}{{M}_\mathrm {med}^2}\\&&\text{ Let } \nu _\lambda {} = \zeta _\lambda {}{M}_\mathrm {med}{}\\ {M}_\mathrm {med}^\mathrm{D1}&= \sqrt{y_\mathrm {q}{}g_\chi {}}\cdot \sqrt{{M}_\star ^3/m_q}&{M}_\mathrm {med}^\mathrm{C1}&= y_\mathrm {q}{}\lambda _\chi {}\zeta _\lambda {}\cdot {M}_\star ^2/m_q \end{aligned}$$

In the above, $\lambda _\chi$ is used for scalar coupling strengths. The vacuum expectation value (VEV) of the trilinear scalar vertex is represented by $\nu _\lambda {}$. The VEV is then related to the mediator mass scale by $\nu _\lambda {}=\zeta _\lambda {}{M}_\mathrm {med}{}$, where the common assumption of $\zeta _\lambda {}\approx 1$ is used. The perturbative range is then $0<\sqrt{y_\mathrm {q}{}g_\chi {}}<4\pi$ for D1 and $0<y_\mathrm {q}{}\lambda _\chi {}\zeta _\lambda {}<(4\pi )^2\zeta _\lambda {}$ for C1.

The operators D11 and C5 describe gluons coupling to WIMPs through a loop diagram, requiring different expressions relating ${M}_\star$ to ${M}_\mathrm {med}$:

$$\begin{aligned}&\mathbf D11&\mathbf C5\\ \frac{\alpha _\mathrm{s}}{4{M}_\star ^3}&= \frac{\alpha _\mathrm{s}g_\chi {}}{{M}_\mathrm {med}^2 \Lambda _\mathrm{s}}&\frac{\alpha _\mathrm{s}}{4{M}_\star ^2}&= \frac{\alpha _\mathrm{s}\lambda _\chi {}\nu _\lambda {}}{{M}_\mathrm {med}^2 \Lambda _\mathrm{s}}\\ {M}_\mathrm {med}{}&= \root 3 \of {\frac{4g_\chi {}}{b}}\,{M}_\star {}&{M}_\mathrm {med}{}&= \root 3 \of {\frac{4\lambda _\chi {}\zeta _\lambda {}}{b}}\,{M}_\star {}\\&\text{ Let } a{}=4b^{-1}&\text{ Let } a{}=4b^{-1}\\ {M}_\mathrm {med}^\mathrm{D11}&= \root 3 \of {a{}g_\chi {}}\,{M}_\star {}&{M}_\mathrm {med}^\mathrm{C5}&= \sqrt{a{}\lambda _\chi {}\zeta _\lambda {}}\,{M}_\star {} \end{aligned}$$

$\Lambda _\mathrm{s} = b{M}_\mathrm {med}{}$ ($b>1$) is another mass suppression scale of the loop connected to the initial-state gluons. The coupling terms differ from the other operators, as $b>1 \ \implies \ 0<a{}<4$. As before, $\nu _\lambda {}=\zeta _\lambda {}{M}_\mathrm {med}{}$, and the assumption of $\zeta _\lambda {}\approx 1$ is used for C5. The perturbative range for the gluon operators is thus $0<\root 3 \of {a{}g_\chi {}}<\root 3 \of {16\pi }$ or $0<\sqrt{a{}\lambda _\chi {}\zeta _\lambda {}}<4\sqrt{\pi \zeta _\lambda {}}$ for D11 and C5 respectively.

A summary of the different relations between ${M}_\mathrm {med}$ and ${M}_\star$ for each operator of interest and the associated coupling ranges is provided in Table 8. All of the operators have a dependence on a coupling term, where the value of these couplings is impossible to know without knowledge of the complete theory. Scans over the coupling-parameter space are therefore performed below to quantify valid phase-space regions.

While these relations were derived for *s*-channel completions, similar validity arguments can be applied to the *t*-channel as a sum of *s*-channel operators (see Ref. [45] for further details and caveats).

**Table 8:** Relations between the mediator mass ${M}_\mathrm {med}$ and the suppression scale ${M}_\star$ for the simplest interaction vertices matching the EFT operators considered here

Operator(s)	Relation between ${M}_\mathrm {med}$ and ${M}_\star$	Coupling term range
D1	${M}_\mathrm {med}{} = \sqrt{y_\mathrm {q}{}g_\chi {}}\ \sqrt{{M}_\star ^3/m_q}$	$0<\sqrt{y_\mathrm {q}{}g_\chi {}}<4\pi$
C1	${M}_\mathrm {med}{} = y_\mathrm {q}{}\lambda _\chi {}\zeta _\lambda {}\ {M}_\star ^2/m_q$	$0<y_\mathrm {q}{}\lambda _\chi {}\zeta _\lambda {}<(4\pi )^2\zeta _\lambda {}$
D5, D8, D9	${M}_\mathrm {med}{} = \sqrt{g_\mathrm {q}{}g_\chi {}}{}\ {M}_\star {}$	$0<\sqrt{g_\mathrm {q}{}g_\chi {}}{}<4\pi$
D11	${M}_\mathrm {med}{} = \root 3 \of {ag_\chi {}}\ {M}_\star {}$	$0<\root 3 \of {ag_\chi {}}<\root 3 \of {16\pi }$
C5	${M}_\mathrm {med}{} = \sqrt{a\lambda _\chi {}\zeta _\lambda {}}\ {M}_\star {}$	$0<\sqrt{a\lambda _\chi {}\zeta _\lambda {}}<4\sqrt{\pi \zeta _\lambda {}}$

### Appendix A.3: Regions of validity

Given a relation between the mediator mass and the suppression scale, $Q_\mathrm{{tr}}{} < {M}_\mathrm {med}{}$ can be evaluated and the fraction of events fulfilling this validity criterion can be determined. Two different procedures are then followed (which were shown to yield the same results). The nominal procedure is a simple truncation, in which the signal cross section is rescaled by the fraction of valid events. With this truncated signal cross section, new valid limits on the mass suppression scale are derived, ${M}_\star ^\mathrm {valid}$.

The second alternative procedure used to cross-check the simple truncation is an iterative procedure that scans through ${M}_\star$ until a convergence point is reached.

1. The starting point is the nominal expected limit on ${M}_\star$ assuming 100 % validity, named ${M}_\star ^\mathrm {exp}{}$. ${M}_\star ^\mathrm {exp}{}$ is set to ${M}_\star ^\mathrm {in}$ before executing step 2 for the first time.
2. For each step *i*, obtain the relative fraction of valid events $R_{{M}_\mathrm {med}{}}^\mathrm {\,i}{}$ satisfying $Q_\mathrm{{tr}}{} < {M}_\mathrm {med}^\mathrm {in}$, where ${M}_\mathrm {med}^\mathrm {in}$ is the mediator mass limit obtained in the previous step (depending on ${M}_\star ^\mathrm {in}$).
3. Truncate ${M}_\star$ following Ref. [44]: $\displaystyle {M}_\star ^\mathrm {out} = [R_{{M}_\mathrm {med}{}}^{\,i}]^{1/2(d-4)}{M}_\star ^\mathrm {in}$, noting that D1 and D11 are dimension $d = 7$ operators, while D5, D8, D9, C1, and C5 are dimension $d = 6$.
4. Go to step 2, using the current ${M}_\star ^\mathrm {out}$ as the new ${M}_\star ^\mathrm {in}$, repeating until the fraction of valid events at a given step $R_{{M}_\mathrm {med}{}}^\mathrm {\,i}{}$ reaches 0 or 1.
5. Calculate the total validity fraction $\displaystyle R_{{M}_\mathrm {med}{}}^\mathrm {\,tot}{} = \prod _i R_{{M}_\mathrm {med}{}}^\mathrm {\,i}{}$ and the truncated limit on the suppression scale $\displaystyle {M}_\star ^\mathrm {valid}{} = [R_{{M}_\mathrm {med}{}}^\mathrm {\,tot}{}]^{1/2(d-4)}{M}_\star ^\mathrm {exp}{}$.

The fraction of valid events and the truncated limits on ${M}_\star$ can be used to assess the validity of the EFT approach. In Figs. 17 and 18, this is shown for D1 and C1. The majority of the parameter space is invalid. The operators D1 and C1 are still valid for regions of parameter space with large coupling values and low WIMP masses.

The validity of the vector, axial-vector, and tensor couplings to quarks via the D5, D8, and D9 operators, respectively, are much more justifiable, as shown for D9 in Fig. 19. The operator D9 is valid for the majority of parameter space, across couplings and WIMP masses, except for the highest values of ${m}_\chi$ considered. While only D9 is valid for the common canonical choice of $g_\mathrm {q}{}=g_\chi {}=1$, the other two operators are valid for only slightly larger couplings.

An assessment of the validity of the gluon EFT operators requires the most assumptions, and has a very different coupling range under the assumptions discussed in Appendix A.2 Under these assumptions, D11 and C5 operators are valid for regions of parameter space with large coupling values and low WIMP masses, as shown in Figs. 20 and 21, respectively.

In general, the validity of the EFT operators is better for low WIMP masses. This is important, as collider searches are most competitive with other types of experiments at low ${m}_\chi$. Additionally, Figs. 17, 18, 19, 20 and 21 show how the truncated limit ${M}_\star ^\mathrm {valid}$ quickly approaches the nominal limit ${M}_\star ^\mathrm {exp}$. Some operators have larger validity regions than others because the ${M}_\star$ limits are larger, and it is thus more likely that $Q_\mathrm{{tr}}{} < {M}_\mathrm {med}{}$. Stronger limits therefore remain strong, while weak limits are in fact even further diminished by validity considerations.

Truncated limits are more conservative than the corresponding simplified model used for the completion, so long as ${M}_\mathrm {med}$ in the model is greater than or equal to the value used for the truncation. This can be seen comparing the D5 operator in Fig. 10 with the corresponding simplified model in Fig. 11.

## Footnotes

1 ATLAS uses a right-handed coordinate system with its origin at the nominal interaction point (IP) in the centre of the detector and the *z*-axis along the beam pipe. The *x*-axis points from the IP to the centre of the LHC ring, and the *y*-axis points upward. The azimuthal angle $\phi$ is measured around the beam axis, and the polar angle $\theta$ is measured with respect to the *z*-axis. We define transverse energy as $E_\mathrm{T} = E \ \mathrm{sin} \theta$, transverse momentum as $p_{\mathrm {T}}= p \ \mathrm{sin} \theta$, and pseudorapidity as $\eta = - \mathrm{ln}[\mathrm{tan}(\theta /2)]$.

2 The charged fraction is defined as $f_{{\text {ch}}}=\sum p_\mathrm{T}^{{\text {track,jet}}} /p_\mathrm{T}^{{\text {jet}}}$, where $\sum p_\mathrm{T}^{{\text {track,jet}}}$ is the scalar sum of the transverse momenta of tracks associated with the primary vertex within a cone of radius $R=0.4$ around the jet axis, and $p_\mathrm{T}^{{ \text {jet}}}$ is the transverse momentum as determined from calorimetric measurements.
